# LRRK2 phosphorylation status and kinase activity regulate (macro)autophagy in a Rab8a/Rab10-dependent manner

**DOI:** 10.1038/s41419-023-05964-0

**Published:** 2023-07-15

**Authors:** Elżbieta Kania, Jaclyn S. Long, David G. McEwan, Kirsten Welkenhuyzen, Rita La Rovere, Tomas Luyten, John Halpin, Evy Lobbestael, Veerle Baekelandt, Geert Bultynck, Kevin M. Ryan, Jan B. Parys

**Affiliations:** 1 Cancer Research UK Beatson Institute, Garscube Estate, Switchback Road, Glasgow, G61 1BD UK; 2 Institute of Cancer Sciences, University of Glasgow, Garscube Estate, Switchback Road, Glasgow, G61 1QH UK; 3 Laboratory of Molecular and Cellular Signaling, Department of Cellular and Molecular Medicine & Leuven Kanker Instituut, KU Leuven, Herestraat 49, Campus Gasthuisberg O&NI – B802, 3000 Leuven, Belgium; 4 Laboratory for Neurobiology and Gene Therapy, Department of Neurosciences & Leuven Brain Institute, KU Leuven, Herestraat 49, Campus Gasthuisberg B1023, 3000 Leuven, Belgium

**Keywords:** Kinases, Macroautophagy

## Abstract

Mutations in the leucine-rich repeat kinase 2 (*LRRK2*) gene are the most common genetic cause of Parkinson’s disease (PD), with growing importance also for Crohn’s disease and cancer. LRRK2 is a large and complex protein possessing both GTPase and kinase activity. Moreover, LRRK2 activity and function can be influenced by its phosphorylation status. In this regard, many LRRK2 PD-associated mutants display decreased phosphorylation of the constitutive phosphorylation cluster S910/S935/S955/S973, but the role of these changes in phosphorylation status with respect to LRRK2 physiological functions remains unknown. Here, we propose that the S910/S935/S955/S973 phosphorylation sites act as key regulators of LRRK2-mediated autophagy under both basal and starvation conditions. We show that quadruple LRRK2 phosphomutant cells (4xSA; S910A/S935A/S955A/S973A) have impaired lysosomal functionality and fail to induce and proceed with autophagy during starvation. In contrast, treatment with the specific LRRK2 kinase inhibitors MLi-2 (100 nM) or PF-06447475 (150 nM), which also led to decreased LRRK2 phosphorylation of S910/S935/S955/S973, did not affect autophagy. In explanation, we demonstrate that the autophagy impairment due to the 4xSA LRRK2 phospho-dead mutant is driven by its enhanced LRRK2 kinase activity. We show mechanistically that this involves increased phosphorylation of LRRK2 downstream targets Rab8a and Rab10, as the autophagy impairment in 4xSA LRRK2 cells is counteracted by expression of phosphorylation-deficient mutants T72A Rab8a and T73A Rab10. Similarly, reduced autophagy and decreased LRRK2 phosphorylation at the constitutive sites were observed in cells expressing the pathological R1441C LRRK2 PD mutant, which also displays increased kinase activity. These data underscore the relation between LRRK2 phosphorylation at its constitutive sites and the importance of increased LRRK2 kinase activity in autophagy regulation and PD pathology.

## Introduction

Mutations in *PARK8*, the gene encoding leucine-rich repeat kinase 2 (LRRK2), have been linked to numerous diseases [1–3]. Specifically, mutations in *PARK8* have been recognized as the most common genetic determinants for familial Parkinson’s disease (PD) [4, 5], but are also associated with sporadic PD [6, 7]. LRRK2 has a complex, domain-organized structure with multiple protein-protein interaction motifs [8–10]. Most interesting, however, is the fact that LRRK2 possesses dual enzymatic activity represented by a kinase domain and a GTPase domain composed of ROC (Ras of complex proteins) and COR-domains (C-terminal of ROC) [8–10].

There are around 100 mutations in LRRK2 described so far, of which eight have been convincingly identified to cause PD. These are point mutations located within the enzymatic core of the protein [11–14]. The common feature of most validated, PD-causative mutants is an increased LRRK2 kinase activity resulting from gain-of-function mutations, such as in the case of the G2019S mutation, which locates within the kinase domain [15]. Interestingly, enhanced kinase activity is also reported for pathological mutants within the GTPase domain, such as R1441C/G/H [16]. The explanation for this perhaps relates to the possibility of crosstalk between the GTPase and kinase domains and their mutual regulation [8, 16, 17].

Another characteristic of LRRK2, is the presence of numerous phosphorylation sites in its structure. These can be divided into two large groups: autophosphorylation sites and sites that are phosphorylated by other kinases, which are also referred to as constitutive phosphorylation sites [18]. Although there is accumulating evidence that LRRK2 phosphorylation is an important parameter for its functioning, the regulation of LRRK2 phosphorylation and the implications of changes in its phosphorylation pattern are only now starting to emerge [19].

The constitutive phosphorylation sites include S910, S935, S955, and S973, located between the ankyrin repeat region and the leucine-rich repeat domain. This cluster of serines can be phosphorylated by cAMP-dependent protein kinase [20, 21], casein kinase-1α [22, 23], or kinases from the IκB family [24]. Apart from enhanced kinase activity, another common feature of several pathological LRRK2 mutants is their -to a greater or lesser extent- decreased levels of phosphorylation at LRRK2 constitutive phosphorylation sites [18, 20, 25–28].

LRRK2 has been previously linked to autophagy regulation, as age-dependent alterations in autophagic markers have been shown in the kidneys of *Lrrk2* knock-out (KO) mice [29]. Dysregulated autophagy was also reported for models harboring the pathological LRRK2 variants G2019S [30–32] and R1441C [33, 34]. Interestingly, LRRK2 S910/S935 phosphorylation-deficient knock-in (KI) mice displayed hallmarks of early PD pathology, including the accumulation of α-synuclein in the striatum and reduced astrocyte numbers [35].

The cellular toxicity, neuronal cell death, and dysregulated autophagy mediated by PD-associated LRRK2 mutants have previously been linked to increased kinase activity of LRRK2 [36, 37]. Yet, it remains unclear which LRRK2-mediated processes are impacted by LRRK2 dephosphorylation at the constitutive sites. Here we address this question with respect to LRRK2-mediated regulation of autophagy under basal and starvation conditions. We report that LRRK2 phosphorylation at constitutive sites is upregulated during starvation. Moreover, we show that the lack of constitutive phosphorylation in an LRRK2 phospho-dead mutant, in which S910/S935/S955/S973 were substituted with alanines (4xSA LRRK2), impairs basal and starvation-induced autophagy. Furthermore, we demonstrate that the 4xSA LRRK2 phospho-dead mutant is characterized by increased kinase activity towards LRRK2 downstream substrates Rab8a and Rab10. Moreover, overexpression of phosphorylation-deficient Rab8a and Rab10 restores the autophagic flux impaired by the presence of 4xSA LRRK2. Enhanced Rab8a and Rab10 phosphorylation was also observed upon expression of the R1441C LRRK2 PD-associated mutant. In addition, cells expressing either 4xSA LRRK2 or R1441C LRRK2 displayed, in a similar manner, impaired basal and starvation-induced autophagy. These findings highlight not only the relevance of LRRK2 phosphorylation status at constitutive sites in autophagy regulation and in PD, but also point out the close connection between LRRK2 phosphorylation status and the kinase function of LRRK2.

## Results

### LRRK2 phosphorylation increases during starvation though not after Torin-1 treatment

**Fig. 1:**
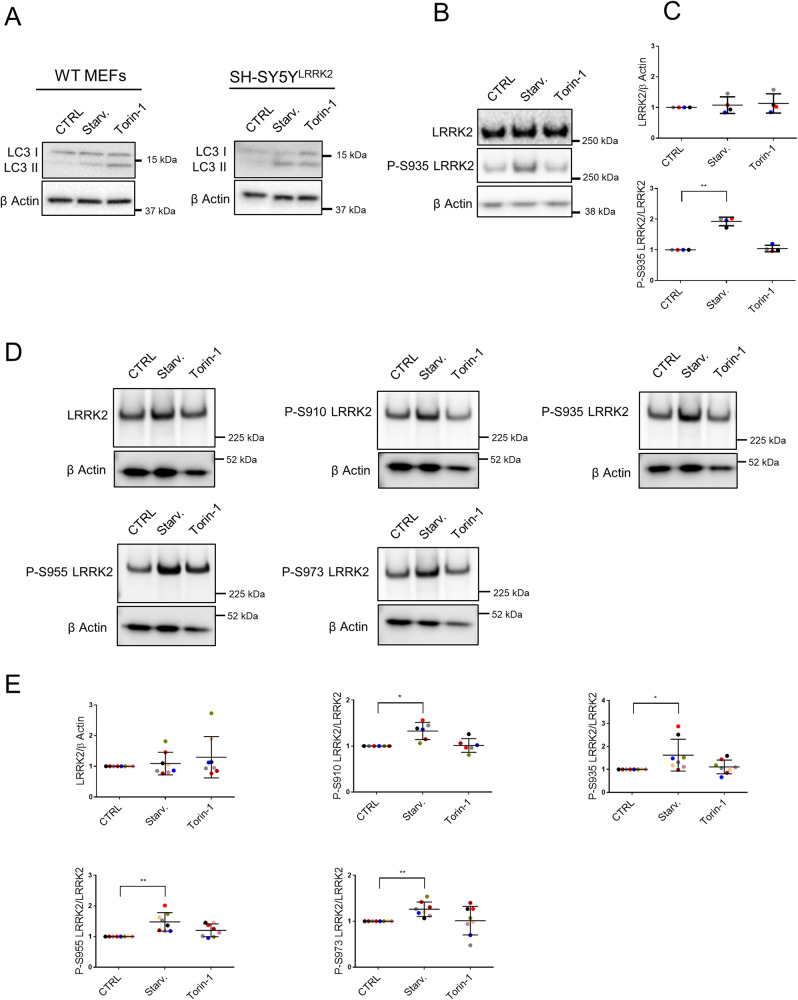
LRRK2 phosphorylation increases during starvation-induced autophagy. WT MEFs and SH-SY5Y^LRRK2^ cells were treated for 2 h with DMSO (CTRL), starvation medium (Starv.), or Torin-1 (50 nM). Representative western blots showing **A** LC3 I and II levels (*N* = 3 independent experiments) in each cell line and **B** LRRK2 expression levels and phosphorylation at S935 in WT MEFs (*N* = 4 independent experiments). **C** Quantification of LRRK2 expression and S935 phosphorylation levels presented in (**B**). **D** Representative western blots showing LRRK2 expression levels and phosphorylation at S910/S935/S955/S973 in SH-SY5Y^LRRK2^ cells (*N* = 6–8 independent experiments). **E** Quantification of LRRK2 expression and phosphorylation levels at S910/S935/S955/S973 presented in (**D**). β actin was used on all blots as a loading control. All graphs are presented as plots of individual data points with mean ± SD, with each experiment marked in a different color and normalized to a control condition (CTRL). Statistical analysis was performed using a Repeated Measures one-way ANOVA with Dunnett’s multiple comparisons post-test (*P < 0.05; **P < 0.001).

We aimed to investigate the involvement of LRRK2 phosphorylation at S910, S935, S955, and S973 during autophagy. Firstly, we treated mouse embryonic fibroblasts (MEFs) with two canonical autophagy inducers: starvation medium (2 h) or Torin-1 (50 nM, 2 h), a mTORC1/2 inhibitor. To validate the effectivity of these treatments, we evaluated the production of microtubule-associated protein 1 A/1B-light chain 3 (LC3) II. In both conditions, increased levels of LC3 II were observed (Fig. 1A). Importantly, we noticed that the phosphorylation of endogenously expressed LRRK2 on S935 was increased two-fold during starvation- but not during Torin-1 treatment, while total LRRK2-expression levels remained unaltered (Fig. 1B, C). Despite the fact that MEFs possess detectable levels of LRRK2, we could not, with the available antibodies, detect phosphorylation at the S910, S955, and S973 sites.

To further analyze LRRK2 phosphorylation in autophagy, we switched to previously described human neuroblastoma SH-SY5Y cells stably overexpressing 3flag-LRRK2 (SH-SY5Y^LRRK2^) [38]. While both starvation and Torin-1 treatment elevated LC3 II levels (Fig. 1A), only starvation increased LRRK2 phosphorylation on all four serines belonging to the phosphorylation cluster (S910/S935/S955/S973) (Fig. 1D, E). Phosphorylation at each of these serine residues increased 1.3 to 1.9-fold, while total LRRK2 protein expression levels remained unaltered (Fig. 1D, E).

To avoid the possible influence of endogenous LRRK2, we also used MEF^hLRRK2^ cells, i.e., MEF KO cells stably overexpressing human WT LRRK2 (Fig. S1A–D). The use of MEF^KO LRRK2^ and MEF^hLRRK2^ cells allowed us to validate the LRRK2 antibodies used (Fig. S1A) and to directly detect the phosphorylation at residues S910, S935, S955, and S973 in the MEF model (Fig. S1C). Similarly to the results above (Fig. 1A), MEF^hLRRK2^ cells also displayed increased LC3 II levels in starvation conditions and after Torin-1 treatment (Fig. S1B), while only starvation increased LRRK2 phosphorylation levels at S910/S935/S955/S973 (Fig. S1C, D). These observations suggest a specific interrelation between LRRK2 phosphorylation at the constitutive sites and the increased LC3 II levels observed during starvation.

### Autophagy is impaired in cell types overexpressing the 4xSA LRRK2 phosphomutant

To investigate the relevance of LRRK2 phosphorylation in starvation-induced autophagy, we used SH-SY5Y cells, which possess very low endogenous LRRK2 protein levels [39], as well as MEF^KO LRRK2^ cells. Both cell lines were transduced with an empty lentiviral vector, or with a vector encoding human WT LRRK2 or 4xSA LRRK2 (the phospho-dead mutant S910A/S935A/S955A/S973A). Importantly, the total LRRK2 expression levels were comparable in SH-SY5Y^LRRK2^ and SH-SY5Y^4xSA LRRK2^ cells (Fig. S2A and Fig. S6B). Moreover, using confocal microscopy, we verified that all SH-SY5Y^LRRK2^ and SH-SY5Y^4xSA LRRK2^ cells in our cell populations expressed the LRRK2 construct (Fig. S2B). In contrast to WT LRRK2-expressing cell models, neither the SH-SY5Y^4xSA LRRK2^ cells (Fig. S2A) nor the MEF^4xSA hLRRK2^ cells (Fig. S2C) showed any phosphorylation at S910, S935, S955, or S973.

**Fig. 2:**
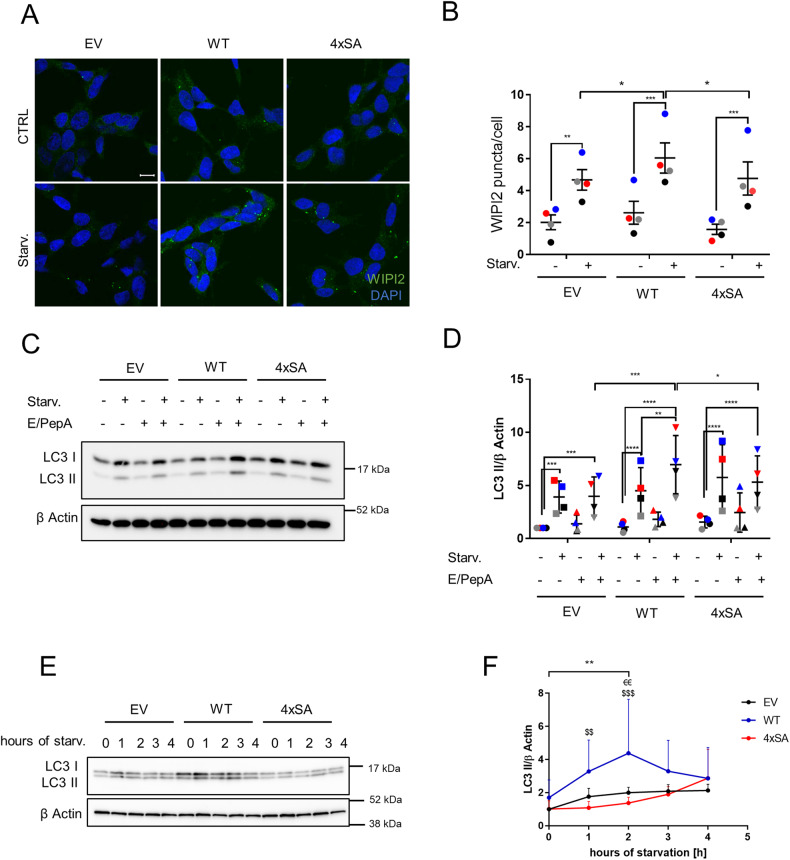
Overexpression of WT LRRK2 but not 4xSA LRRK2 phosphomutant stimulates starvation-induced autophagy. **A**, **B** SH-SY5Y^EV^, SH-SY5Y^LRRK2^, and SH-SY5Y^4xSA LRRK2^ cells remained untreated (CTRL) or were treated for 2 h with starvation medium (Starv.). **A** Representative images showing WIPI2 puncta from *N* = 4 independent experiments with at least 1200 cells analysed per condition. Scale bar: 20 μm. **B** Quantification of the WIPI2 puncta per cell shown in (**A**). The means of all experiments ± SEM are shown with each experiment marked in a different color. For statistical analysis, a Repeated Measures two-way ANOVA with Sidak’s multiple comparisons post-test (*P < 0.05; **P < 0.01; ***P < 0.001) was used. **C**, **D** SH-SY5Y^EV^, SH-SY5Y^LRRK2^, and SH-SY5Y^4xSA LRRK2^ cells were treated for 2 h with DMSO or starvation medium (Starv.) in the absence or presence of the lysosomal protease inhibitors E64d and pepstatin A (E/PepA; 10 μM each). **C** Representative western blot showing LC3 I and II levels (*N* = 4 independent experiments). β actin was used as a loading control. **D** Quantification of the LC3 II levels presented in (**C**) shown as a plot of the individual data points with mean ± SD with each experiment marked in a different color and normalized to the empty vector CTRL (no starvation medium, no protease inhibitors). Repeated Measures two-way ANOVA with Sidak’s multiple comparisons post-test (*P < 0.05; **P < 0.01; ***P < 0.001; ****P < 0.0001) was used for statistical analysis. **E**, **F** MEF^EV^, MEF^hLRRK2^, and MEF^4xSA hLRRK2^ cells remained untreated (CTRL) or were treated with starvation medium (Starv.) for 1, 2, 3 and 4 h. **E** Representative western blot showing LC3 levels with β actin as loading control (*N* = 3 independent experiments). **F** Quantification of the LC3 II levels during the starvation time course presented in (**E**) shown as mean ± SD (*N* = 3 independent experiments) and normalized to the empty vector CTRL (no starvation medium). Repeated Measures two-way ANOVA with Sidak’s multiple comparisons post-test was used for statistical analysis: **P < 0.01 indicates differences for MEF^hLRRK2^ cells only; ^€€^*P* < 0.01 marks the difference between MEF^hLRRK2^ and MEF^EV^ cells; ^$$^*P* < 0.01 and ^$$$^*P* < 0.001 marks the difference between MEF^hLRRK2^ and MEF^4xSA hLRRK2^ cells.

Next, we evaluated the occurrence of autophagy in either replete or starvation culture medium. The WD repeat domain, phosphoinositide interacting protein 2 (WIPI2), serves as a marker for omegasome formation, an early autophagy hallmark [40]. Using immunofluorescent imaging, we observed in SH-SY5Y^LRRK2^ cells that the increase in number of WIPI2 puncta per cell during starvation was significantly higher than in SH-SY5Y^4xSA LRRK2^ or in SH-SY5Y^EV^ (containing an empty vector) cells (Fig. 2A, B). In addition, there was no significant increase in WIPI2 puncta during starvation-induced autophagy in MEF^4xSA hLRRK2^ cells (Fig. S3A, B).

To evaluate autophagic flux, we compared the autophagic response in the absence or presence of the lysosomal protease inhibitors E64d and pepstatin A (E/PepA), which block autophagic degradation (Fig. 2C). In SH-SY5Y^LRRK2^ cells, we observed a 4.5-fold increase in LC3 II levels after starvation alone and a further, statistically significant increase (to 7-fold) when starvation and E/PepA treatment were combined (Fig. 2D). Moreover, in SH-SY5Y^EV^ and SH-SY5Y^4xSA LRRK2^ cells, LC3 II accumulation was in the presence of E/PepA not greater than after starvation alone, pointing towards retarded starvation-induced autophagic flux (Fig. 2C, D). Starvation-induced autophagic flux was also impaired in SH-SY5Y^4xSA LRRK2^ cells by the lysosomotropic agent chloroquine (Fig. S3C).

Finally, we also evaluated autophagy in the MEF model. When assessing LC3 II levels during starvation for up to 4 h, we could only observe a significant increase in LC3 II levels after 2 h of starvation in the MEF^hLRRK2^ cells, followed by a decrease in LC3 II levels at later time points. This indicates a complete autophagic flux, which was not observed in MEF^EV^ or MEF^4xSA LRRK2^ (Fig. 2E, F).

These results indicate that in SH-SY5Y cells and in MEFs, overexpression of WT LRRK2, but not of the 4xSA LRRK2 phospho-dead mutant, enhances autophagy induction and autophagic flux.

### LRRK2 kinase inhibitors neither impact autophagy initiation nor overall autophagic flux

Next, we examined whether pharmacological inhibition of LRRK2 kinase activity could interfere with autophagy initiation. It was previously described [38, 41, 42] that treatment with various LRRK2 kinase inhibitors decreased the phosphorylation level of each of the four serines of the phosphorylation cluster. Therefore, we first validated whether 2 h treatment with MLi-2 (100 nM) or PF-06447475 (150 nM), two very potent and specific LRRK2 kinase inhibitors [43, 44], was sufficient to counteract their phosphorylation (Fig. S4). As total LRRK2 levels were unchanged (Fig. S4), the decreased phosphorylation at these serines was not caused by LRRK2 degradation [38].

**Fig. 3:**
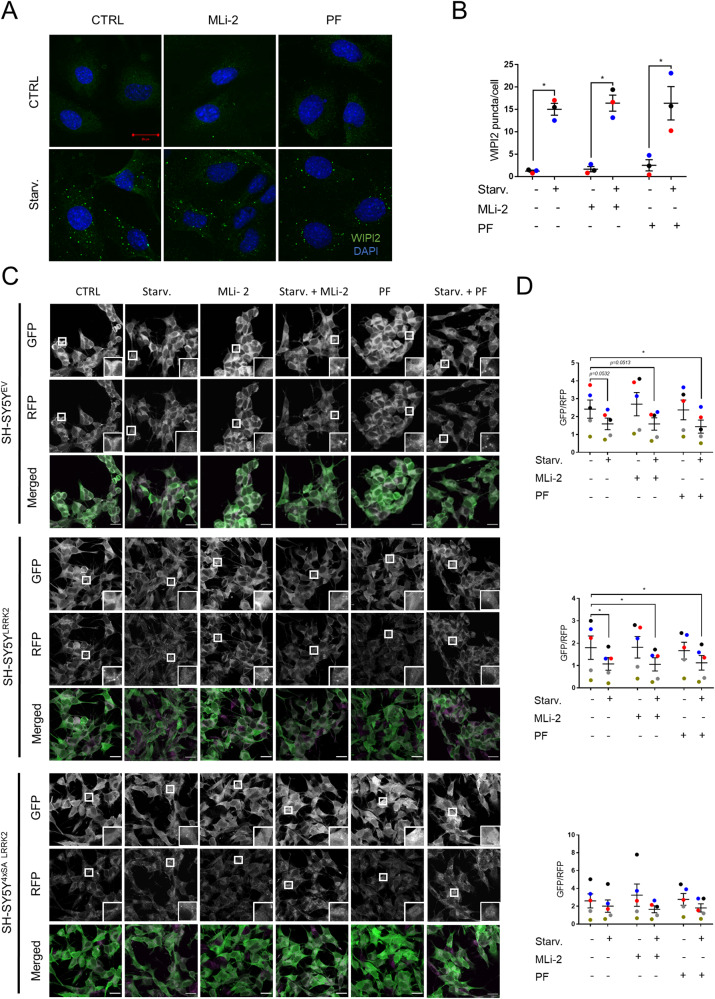
LRRK2 kinase inhibition does neither impact the early stages of autophagy nor autophagic degradation. **A** Representative images showing WIPI2 puncta (green) and nuclei stained with DAPI (blue) in WT MEFs treated for 2 h with DMSO (CTRL) or starvation medium (Starv.), in the absence or presence of the LRRK2 kinase inhibitors MLi-2 (100 nM) or PF-06447475 (PF, 150 nM). *N* = 3 independent experiments with at least 500 cells analysed per condition. Scale bar = 20 μm. **B** Quantification of the WIPI2 puncta per cell shown in (**A**). The means of each experiment ± SEM are shown; Repeated measures two-way ANOVA with Sidak’s multiple comparisons post-test (*P < 0.05) was used for statistical analysis. **C**, **D** SH-SY5Y^EV^, SH-SY5Y^LRRK2^, and SH-SY5Y^4xSA LRRK2^ cells expressing the GFP-LC3-RFP-LC3ΔG construct were treated for 4 h with DMSO (CTRL) or starvation medium (Starv.), in the absence or presence of MLi-2 (100 nM) or of PF-06447475 (PF, 150 nM). **C** Representative images of GFP-LC3-RFP-LC3ΔG expression are shown; scale bar = 20 μm. **D** Quantification of the autophagic degradation process, measured as the GFP/RFP ratio and presented as a plot of individual data points with mean ± SEM. From top to bottom, the graphs correspond to the three types of cell (SH-SY5Y^EV^, SH-SY5Y^LRRK2^, and SH-SY5Y^4xSA LRRK2^) as presented in (**C**); *N* = 5 independent experiments, each involving 6 technical replicates. Statistical analysis was performed using a Repeated Measures two-way ANOVA with Sidak’s multiple comparisons post-test (*P < 0.05).

Treatment with these inhibitors did not affect starvation-induced WIPI2 puncta formation in WT MEFs (Fig. 3A, B). To accurately measure autophagic flux, we used the GFP-LC3-RFP-LC3ΔG probe [45]. This probe is intracellularly cleaved to equimolar amounts of GFP-LC3, which is degraded by autophagy, and RFP-LC3ΔG, which serves as an internal control. Therefore, the GFP/RFP ratio accurately reflects autophagic flux. We compared SH-SY5Y^EV^, SH-SY5Y^LRRK2^, and SH-SY5Y^4xSA LRRK2^ cells under basal and under starved conditions, in the absence or presence of MLi-2 or PF-06447475 (Fig. 3C, D). The decreased GFP/RFP ratio observed in starved cells corresponds to LC3 degradation and, thus, a proficient autophagic flux (Fig. 3D). Starvation increased autophagic flux in empty vector-transduced cells and in cells overexpressing WT LRRK2. However, no autophagic flux could be observed in the SH-SY5Y^4xSA LRRK2^ cells. Treatment with either MLi-2 or PF-06447475 by itself had no effect on either basal or starvation-induced autophagic flux.

In summary, pharmacological LRRK2 inhibition leads to decreased phosphorylation on S910, S935, S955, and S973, yet did not affect the propensity of SH-SY5Y cells to undergo basal or starvation-induced autophagy.

### Reduced markers and functionality of the acidic compartments in cells overexpressing the 4xSA LRRK2 phosphomutant

Next, to understand the difference in autophagic response between cells expressing WT and 4xSA LRRK2, we examined the endo-lysosomal compartments in these cells.

**Fig. 4:**
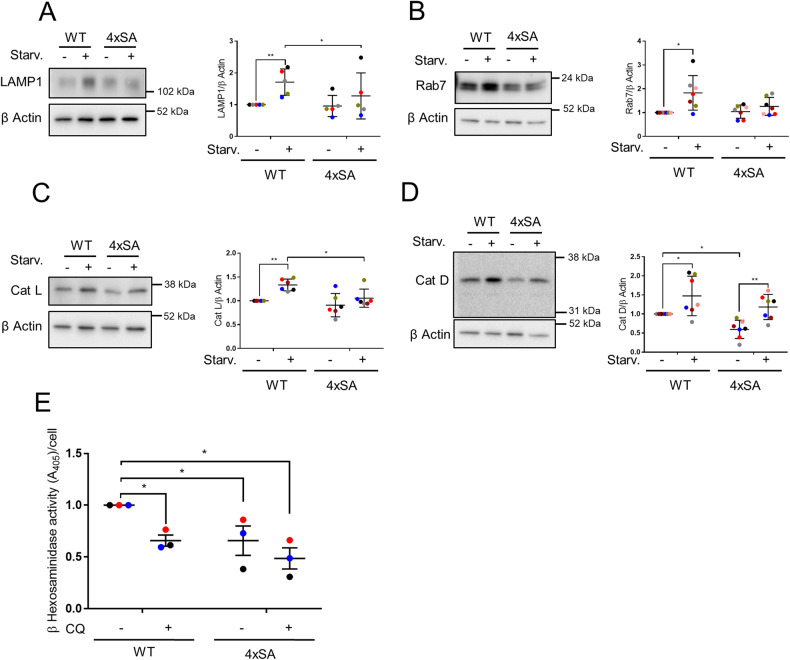
Levels of lysosomal/endosomal markers and enzymes are lower in SH-SY5Y^4xSA LRRK2^ than in SH-SY5Y^LRRK2^ cells. **A**–**D** SH-SY5Y^LRRK2^ and SH-SY5Y^4xSA LRRK2^ cells remained untreated (CTRL) or were treated for 2 h with starvation medium (Starv.). Representative western blots showing the levels of (**A**) LAMP1 (*N* = 5 independent experiments), **B** Rab7 (*N* = 7 independent experiments), **C** Cathepsin L (Cat L) (*N* = 6 independent experiments), and **D** Cathepsin D (Cat D) (*N* = 7 independent experiments). β actin was used as a loading control. The graphs show the quantification of resp. LAMP1, Rab7, Cat L, and Cat D levels relative to the loading control, presented as a plot of individual data points with mean ± SD with each experiment marked in a different color and normalized to the control condition (WT LRRK2, no starvation); Repeated Measures two-way ANOVA with Sidak’s multiple comparisons post-test (*P < 0.05; **P < 0.01). **E** Graph showing the activity of β-hexosaminidase measured in SH-SY5Y^LRRK2^ and SH-SY5Y^4xSA LRRK2^ cells treated for 2 h with or without chloroquine (CQ, 10 μM). The graph is presented as individual data points with mean ± SEM normalized to the control condition (WT LRRK2, no CQ); *N* = 3 independent experiments with 3 technical replicates per experiment. Statistical significance is shown in comparison to the control condition; Repeated Measures two-way ANOVA with Dunnett’s multiple comparisons post-test (*P < 0.05).

Starvation for 2 h provoked the upregulation of lysosomal-associated membrane protein 1 (LAMP1) and of the late endosomal marker Rab7 in SH-SY5Y^LRRK2^ but not in SH-SY5Y^4xSA LRRK2^ cells (Fig. 4A, B). We subsequently investigated whether the lysosomes in SH-SY5Y^4xSA LRRK2^ cells have normal levels of degradative enzymes. The levels of the mature form of cathepsin L (Cat L) were significantly elevated in starvation conditions in SH-SY5Y^LRRK2^ cells and were higher than in SH-SY5Y^4xSA LRRK2^ cells (Fig. 4C). Protein levels of mature cathepsin D (Cat D) were significantly increased by starvation in both SH-SY5Y^LRRK2^ and SH-SY5Y^4xSA LRRK2^ cells (Fig. 4D). Moreover, the SH-SY5Y^4xSA LRRK2^ cells exhibit a significantly decreased (−35%) activity of β-hexosaminidase (Fig. 4E). Chloroquine was used in this experiment as a pH-neutralizing agent, significantly impairing β-hexosaminidase activity (Fig. 4E) [46]. Finally, we observed that the Cat D levels, as well as the β-hexosaminidase activity, were under basal conditions significantly lower in the SH-SY5Y^4xSA LRRK2^ cells when compared to the SH-SY5Y^LRRK2^ cells (Fig. 4D, E). This decrease in lysosomal enzyme activity was, however, neither due to a general decline in lysosome number nor to their lesser acidity (Fig. S5), suggesting that the S910A/S935A/S955A/S973A mutation in LRRK2 affects lysosomal enzymes in a rather more direct way than by changing global lysosomal biology.

These data demonstrate that after induction of starvation, the activity of the acidic compartments is reduced in cells overexpressing 4xSA LRRK2 compared to those overexpressing WT LRRK2.

### 4xSA LRRK2-mediated phosphorylation of Rab8a and Rab10 is instrumental for the retardation of the autophagic flux

**Fig. 5:**
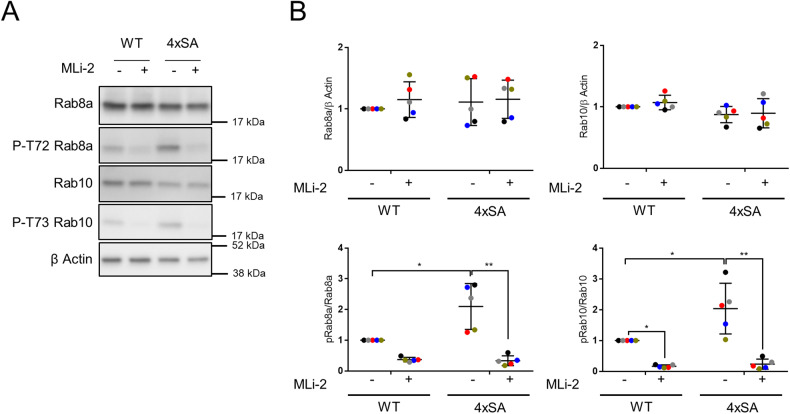
SH-SY5Y^4xSA LRRK2^ cells have increased LRRK2 kinase activity towards Rab8a and Rab10. SH-SY5Y^LRRK2^ and SH-SY5Y^4xSA LRRK2^ were treated for 2 h with DMSO or with the LRRK2 kinase inhibitor MLi-2 (100 nM). **A** Representative western blots showing the levels of total Rab8a, total Rab10, and the phosphorylation levels of Rab8a at T72 and of Rab10 at T73 (*N* = 5 independent experiments). β actin was used as a sample integrity control. **B** Quantification of the levels of total and phosphorylated Rab8a and Rab10 levels presented in (**A**). Graphs are presented as plots of individual data points with mean ± SD, with each experiment marked in a different color and normalized to the control condition (WT LRRK2, no MLi-2). Statistical significance is shown in comparison to the control condition (SH-SY5Y^LRRK2^ cells in the absence of MLi-2 treatment); statistical analysis was performed using a Repeated Measures two-way ANOVA with Sidak’s multiple comparisons post-test (*P < 0.05; **P < 0.01).

To understand the differences in the autophagic process between the lack of S910/S935/S955/S973 phosphorylation due to LRRK2 inhibitors and to 4xSA LRRK2 expression, we evaluated the LRRK2 kinase activity in SH-SY5Y^LRRK2^ and SH-SY5Y^4xSA LRRK2^ cells, in absence or presence of MLi-2 (100 nM). We, therefore, assessed the phosphorylation of Rab8a at T72 and of Rab10 at T73, two canonical downstream targets of LRRK2 kinase [47–49]. While 2 h MLi-2 treatment leads to a nearly full inhibition of Rab8a and Rab10 phosphorylation in both cell lines, their basal phosphorylation levels were significantly higher in the phosphomutant cells (Fig. 5A, B). These findings reveal opposing effects of MLi-2, which inhibits LRRK2-kinase activity, versus the 4xSA LRRK2 phospho-dead mutant, which augments LRRK2-kinase activity.

**Fig. 6:**
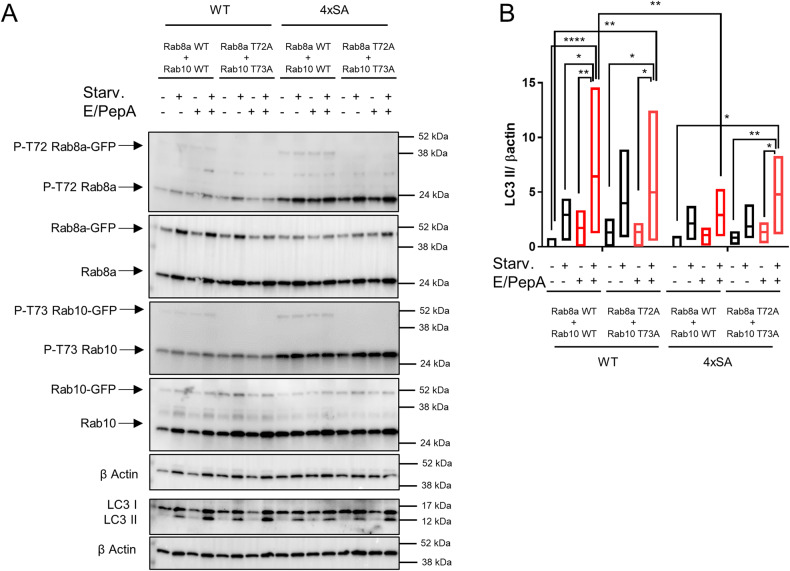
Ectopic expression of phosphorylation-deficient T72A Rab8a and T73A Rab10 restores starvation-induced autophagic flux in SH-SY5Y^4xSA LRRK2^ cells. SH-SY5Y^LRRK2^ and SH-SY5Y^4xSA LRRK2^ cells were co-transfected with either pDEST53-GFP-Rab8a together with pDEST53-GFP-Rab10 or with pDEST53-GFP-Rab8aT72A together with pDEST53-GFP-Rab10T73A. Cells were treated for 2 h with DMSO or starvation medium (Starv.) in the absence or presence of lysosomal proteases inhibitors E64d and pepstatin A (E/PepA; 10 μM each). **A** Representative western blots show the levels of total Rab8a, total Rab10, and the levels of P-T72 Rab8a and of P-73 Rab10 as well as of LC3 I and LC3 II (*N* = 3–5 independent experiments). β actin was used as a sample integrity control. **B** Quantification of the LC3 II levels presented in (**A**) normalized to the empty vector CTRL (no starvation medium, no protease inhibitors). Floating bars show the minimum and maximum values for each condition, with the line indicating the mean (*N* = 5 independent experiments). Samples treated with E64d and pepstatin A are shown in red. Repeated Measures two-way ANOVA with Sidak’s multiple comparisons post-test (*P < 0.05; **P < 0.01; ****P < 0.0001) was used for statistical analysis.

Next, we wondered whether the decreased autophagic flux after overexpression of 4xSA LRRK2 was due to the increased phosphorylation of Rab8a and Rab10. To test this, we expressed GFP-tagged phospho-dead mutants T72A Rab8a and T73A Rab10 or their WT equivalents in SH-SY5Y^LRRK2^ and SH-SY5Y^4xSA LRRK2^ cells. Expression of T72A Rab8a and T73A Rab10, but not of their WT counterparts, restored autophagic flux in SH-SY5Y^4xSA LRRK2^ cells (Fig. 6A, B). Hence, the increased LRRK2 kinase activity and the subsequent increased phosphorylation of Rab8a and Rab10 play a crucial role in the impairment of autophagic flux in SH-SY5Y^4xSA LRRK2^ cells.

### 4xSA LRRK2 displays functional similarities to R1441C LRRK2

The R1441C LRRK2 mutation, located within the GTPase domain, is a PD-related mutation associated with increased kinase activity [50]. Phosphorylation activity towards Rab8a and Rab10 increased in SH-SY5Y^R1441C LRRK2^ cells to a similar extent as in the SH-SY5Y^4xSA LRRK2^ phospho-dead mutant cells, while it is nearly completely abolished by MLi-2 treatment (Fig. S6A). Moreover, after compensation for expression level, SH-SY5Y^R1441C LRRK2^ cells show lower LRRK2 phosphorylation levels at S910/S935/S955/S973 than SH-SY5Y^LRRK2^ cells (Fig. S6B, C). A lower level of LRRK2 phosphorylation at S935 was also observed in MEF^R1441C LRRK2^ (Fig. S6D).

**Fig. 7:**
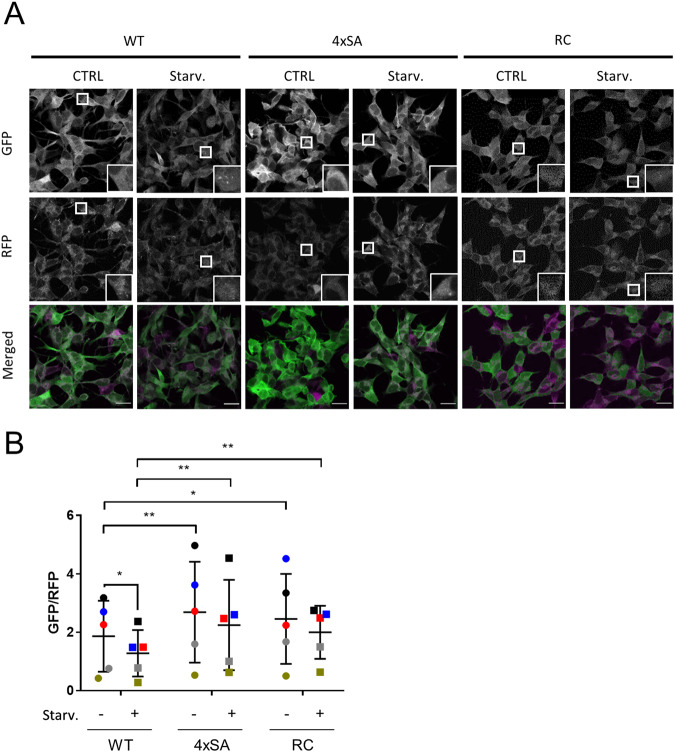
SH-SY5Y^4xSA LRRK2^ and SH-SY5Y^R1441C LRRK2^ cells demonstrate a similarly impaired basal and starvation-induced autophagic flux. SH-SY5Y^LRRK2^, SH-SY5Y^4xSA LRRK2^, and SH-SY5Y^R1441C LRRK2^ cells expressing the GFP-LC3-RFP-LC3ΔG construct were analysed for autophagic degradation by measuring the GFP/RFP ratio. The cells were treated with DMSO (CTRL) or with starvation medium (Starv.) for 2 h. **A** Representative image showing GFP-LC3-RFP-LC3ΔG expression; scale bar = 20 μm. **B** Quantification of autophagic degradation measured as GFP/RFP ratio is presented as a plot of individual data points with mean ± SEM; *N* = 5 independent experiments each involving three to six technical replicates. Statistical analysis was performed using a Repeated Measures two-way ANOVA with Sidak’s multiple comparisons post-test (*P < 0.05; **P < 0.01). Please note that the data shown in (**B**) form part of the data set also presented in Fig. 3D.

Lastly, given that 4xSA LRRK2, as well as R1441C LRRK2, show a reduced phosphorylation of the serines of the phosphorylation cluster, but a higher LRRK2 kinase activity, we analyzed autophagic flux in SH-SY5Y cells overexpressing these mutants (Fig. 7A). By using the GFP-LC3-RFP-LC3ΔG probe and fluorescence imaging we observed impaired basal autophagy in both cell lines (Fig. 7B). Moreover, in SH-SY5Y^4xSA LRRK2^ (as also shown in Fig. 3D) or in SH-SY5Y^R1441C LRRK2^ cells, the degradation of GFP-LC3 was largely impaired under starvation conditions. These data, therefore, show that autophagic flux is impaired under both basal and starvation conditions in both 4xSA LRRK2 and PD-associated R1441C LRRK2-expressing cells.

## Discussion

For this study, we employed four independent cell models to examine the role of LRRK2 phosphorylation in the control of autophagy. First, we used SH-SY5Y cells stably overexpressing either WT or mutant LRRK2. SH-SY5Y cells express endogenous LRRK2 at very low levels [39]; therefore, the stable cell lines form a widely used model for the study of the functional effects of LRRK2 in a neurodegenerative context [38, 41]. We also used WT MEFs, which express LRRK2 at a measurable level, as well as MEFs derived from R1441C LRRK2 KI mouse. Finally, in order to study the functional effects of LRRK2 in the absence of any possible interference by its endogenous levels and because not all antibodies used sufficiently recognize murine LRRK2, we also included an LRRK2 KO model [51]. In this KO model, we re-introduced either human WT or mutant LRRK2.

LRRK2 has been linked to the regulation of many cellular processes, including cell death [52, 53], cell proliferation [54, 55], vesicular trafficking, and autophagy [9, 56, 57]. Here we demonstrate the importance of phosphorylation of LRRK2 at its constitutive sites (S910, S935, S955, and S973) with regard to autophagy regulation.

In starvation conditions, phosphorylation of LRRK2 at S910, S935, S955, and S973 is promoted in both MEFs and SH-SY5Y cells (Fig. 1B–E and Fig. S1C, D). The phosphorylation status of LRRK2 might impact its cellular functions by regulating LRRK2’s interaction with 14-3-3 proteins, LRKK2’s cellular distribution, and its role in response to stress [18]. In contrast, when S910 and S935 are dephosphorylated, LRRK2 can interact with the E3 ubiquitin ligase TRIM1, leading to the microtubular recruitment of LRRK2, its ubiquitination and proteasomal degradation, while also preventing Rab29-mediated LRRK2 activation [58]. LRRK2 phosphorylation at S935 and subsequent membrane recruitment has been previously observed in LPS-activated RAW264.7 and BV2 monocytes, and this preceded autophagy induction [59]. On the other hand, it has also been shown that in response to arsenite stress, LRRK2 undergoes dephosphorylation at S910 and S935, which resulted in loss of the LRRK2-14-3-3 connection, LRRK2 self-association and translocation to the centrosomes [60]. The impact of these events on cellular functions exerted by LRRK2, however, is not yet understood.

In this study, the absence of LRRK2 phosphorylation at constitutive sites in cells featuring the 4xSA LRRK2 phospho-dead mutant, resulted in a potent blockage of autophagy (Fig. 2A–F, Fig. S3A–C, and Fig. 7A, B). In addition, 4xSA LRRK2 phospho-dead mutant cells are characterized by lower levels of lysosomal markers and degradative enzymes than the cells overexpressing WT LRRK2 (Fig. 4A–E). These lower levels did, however, neither correlate with a lower number of lysosomes nor with an increased lysosomal pH (Fig. S5A, B).

Interestingly, LRRK2 mutants in which S860, S910, S935, S955, S973, and S976 were all mutated to alanines (6xSA LRRK2) or aspartates (6xSD LRRK2) have been recently developed [61]. When expressed in HEK293T cells, 6xSA LRRK2 maintained its phosphorylation activity towards Rab8a and Rab10, but 6xSD LRRK2 showed strongly decreased phosphorylation properties. Moreover, Rab29-stimulated LRRK2 autophosphorylation at S1292 was 6x higher for 6xSA LRRK2 than for 6xSD LRRK2. With respect to lysosomal properties, PC12 pheochromocytoma cells expressing either 6xSA or 6xSD LRRK2 demonstrated a slight reduction in the number of lysosomes, which could be rescued by treatment with chloroquine, though there was no differences in level of lysosomal glucocerebrosidase activity.

WIPI2 is a phosphatidylinositol-3-phosphate (PtdIns3P) adapter protein, and decreased numbers of WIPI2 puncta were observed in 4xSA LRRK2-expressing cells (Fig. 2A, B and Fig. S3A, B). WIPI2 is essential for the recruitment of the ATG15-ATG12-ATG16L1 E3-like complex that drives LC3 lipidation onto autophagy membranes and could account for the decreased LC3 II levels observed [40]. These observations support the role of LRRK2 phosphorylation at constitutive sites in autophagy regulation.

Kinase inhibitors exist in general in two types: type 1 inhibitors that target the ATP-binding site of the kinase in its active conformation and type 2 inhibitors that target a site available in the inactive conformation [62]. Dephosphorylation of LRRK2 at constitutive sites was previously shown to occur following treatment with various LRRK2 kinase inhibitors, all belonging to the type 1 group [38, 41, 42]. We demonstrated that MLi-2 and PF-06447475, two specific LRRK2 type 1 kinase inhibitors, resulted in dephosphorylation of S910, S935, S955, and S973 (Fig. S4). Type 2 inhibitors, however, do not affect S910, S935, S955, and S973 phosphorylation levels, probably by stabilizing a conformation of LRRK2, whereby the constitutive phosphorylation cluster remains accessible for kinases and/or inaccessible for phosphatases [63]. In contrast to the 4xSA LRRK2 phosphomutant, however, MLi-2 and PF-06447475 neither affected the initiation of autophagy as assessed by measuring WIPI2 puncta formation (Fig. 3A, B) nor autophagic turnover measured using the GFP-LC3-RFP-LC3ΔG probe (Fig. 3C, D). MLi-2 and PF-06447475 did also neither affect LC3 II levels nor lysosomal protein degradation in cultured primary cortical neurons bearing the R1441C LRRK2 mutation [33]. However, LRRK2 inhibitors were shown to modulate autophagy in other PD-related cellular models [64]. MLi-2 decreased lysosomal pH in β-glucocerebrosidase 1 mutant astrocytes [65], while PF-06447475 boosted lysosome numbers and their activity as well as autolysosome formation and α-synuclein clearance in G2019S-expressing SH-SY5Y cells [39].

In contrast to the MLi-2 or PF-06447475-mediated LRRK2 inhibition, LRRK2 kinase activity towards its known downstream targets Rab8a and Rab10 was significantly higher in SH-SY5Y^4xSA LRRK2^ cells (Fig. 5A, B). Interestingly, mild lysosomal damage evoked by pathogens or by lysosomotropic drugs leads to LRRK2 translocation to the lysosomes and subsequent phosphorylation of various members of the small Rab GTPase family. A higher level of phosphorylation of Rab8a at T72 and/or Rab10 at T73 was also observed after treatment of macrophages and HEK293 cells with chloroquine [47, 66], with various pathogens or with the lysosomal membrane-rupturing agent L-leucyl-L-leucinemethyl ester (LLOMe) [67]. Similarly, in primary astrocytes exposed to LLOMe, increased Rab10 and Rab35 phosphorylation was observed after the recruitment of LRRK2 to the lysosomes [68]. Although, depending on the applied stress and the cell type concerned, the phosphorylation levels of the various Rab proteins may differ, they always promote lysosomal homeostasis [47, 67, 68]. Finally, we should mention that Rab10 phosphorylation was suppressed in MEFs derived from S910A/S935A KI mice [69]. A subsequent study on the same S910A/S935A KI mice confirmed the suppression of Rab10 phosphorylation in kidney tissue but not in the brain, lung, or spleen, which may be due to the low LRRK2 expression levels in the latter tissues [35]. The strong increase in Rab8a and Rab10 phosphorylation we observed in SH-SY5Y^4xSA LRRK2^ can be due to the fact that four serines of the constitutive phosphorylation cluster are mutated to alanines instead of two, though we cannot completely exclude cell type-specific effects.

The retarded autophagic flux observed in cells expressing 4xSA LRRK2 and the increased kinase activity of this mutant converge at the increased phosphorylation levels of the small G proteins Rab8a and Rab10. Indeed, expression of the dominant-negative phospho-dead mutants T72A Rab8a and T73A Rab10 was sufficient to restore the retarded autophagic flux in SH-SY5Y^4xSA LRRK2^ cells (Fig. 6A, B), suggesting a crucial role for Rab8a and Rab10 phosphorylation in the process.

**Fig. 8:**
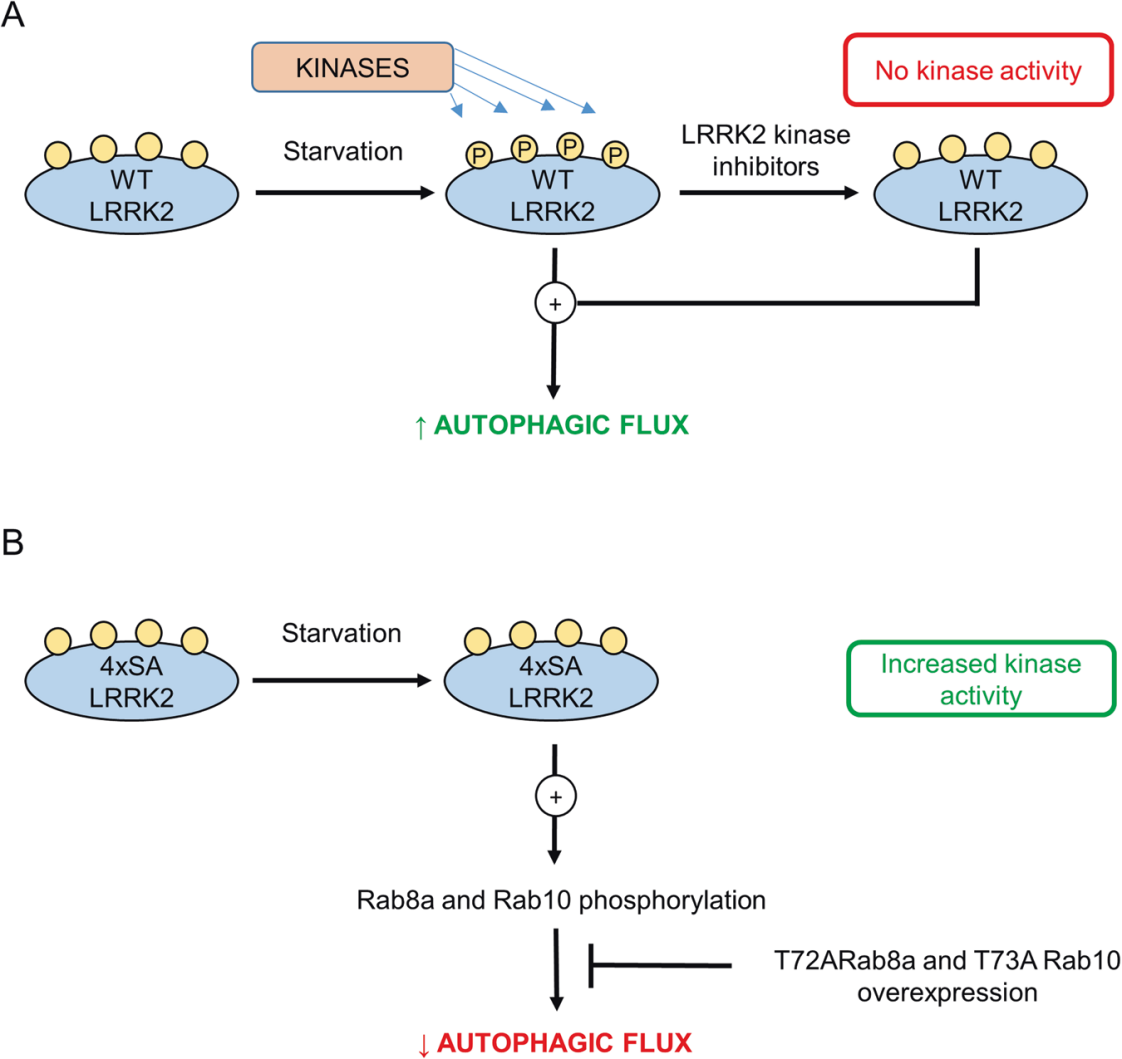
Model of autophagy regulation by LRRK2 phosphorylation at S910/S935/S955/S973 during starvation. **A** During starvation, WT LRRK2 is extensively phosphorylated at S910/S935/S955/S973, stimulating the autophagic process. Treatment with the LRRK2 kinase inhibitors MLi-2 or PF-06447475 inhibits the LRRK2 kinase activity and leads to S910/S935/S955/S973 dephosphorylation. **B** Substitution of the serines with alanines (S910A/S935A/S955A/S973A; 4xSA LRRK2) prohibits their phosphorylation, also under starvation conditions. However, in contrast to what happens during treatment with LRRK2 inhibitors, 4xSA LRRK2 is characterized by increased kinase activity. Due to the resulting increased downstream phosphorylation of Rab8a and Rab10, autophagic flux is impaired. The latter can be rescued by overexpression of phospho-dead mutants T72A Rab8a and T73A Rab10. Color code: green, stimulation; red, inhibition.

These findings allow us to propose the following model (Fig. 8). During starvation, LRRK2 is extensively phosphorylated at the constitutive phosphorylation sites S910/S935/S955/S973 and this relates to increased autophagic flux. Treatment with specific LRRK2 inhibitors leads to kinase inhibition and dephosphorylation of LRRK2. However, no effects on autophagy can be observed. Substitution of the above-mentioned serines with alanines (4xSA LRRK2) makes phosphorylation at these sites impossible. This mutant, however, displays a higher LRRK2 kinase activity, leading to higher levels of phosphorylated Rab8a and Rab10. As a result, cells overexpressing 4xSA LRRK2 form less WIPI2-positive structures during starvation compared to cells expressing WT LRRK2, and autophagic flux is retarded. Importantly, this autophagy impairment can be rescued by the expression of phosphorylation-defective T72A Rab8a and T73A Rab10 mutants, underscoring the role of LRRK2-mediated Rab8a and Rab10 phosphorylation in the process.

Finally, increased LRRK2 kinase activity is a characteristic of several PD-associated LRRK2 mutants and is believed to underlie their disease-promoting effects [36, 37]. As Rab29 promotes the recruitment of LRRK2 to the trans-Golgi network (TGN) and stimulates its kinase activity [42], an enhanced interaction of dephosphorylated LRRK2 with Rab29 and/or a more prominent recruitment of LRRK2 to the TGN could possibly explain the higher kinase activity in 4xSA LRRK2. In support of this, an increased stimulation by Rab29 was recently reported for 6xSA LRRK2 [61]. In addition, Rab29-associated LRRK2 activation and TGN recruitment is more evident in R1441C/G and Y1699C LRRK2 variants, which highlights similarities to our 4xSA LRRK2 model.

Reduced LRRK2 phosphorylation at S910/S935/S955/S973, followed by enhanced LRRK2 kinase activity, could, in fact, be considered as part of a pathological mechanism leading to PD. Intriguingly, LRRK2 dephosphorylated at the constitutive phosphorylation sites is observed in most pathogenic mutations and can as well be found in the substantia nigra pars compacta of idiopathic PD patients [70]. Moreover, PD-associated LRRK2 mutants such as I2020T [71] and R1441C [72] that show reduced phosphorylation at the constitutive sites [23, 60] are also more prone to degradation compared with WT LRRK2. We also confirmed the decreased LRRK2 expression levels in cells expressing the R1441C mutant and their relatively lower level of phosphorylation at the constitutive phosphorylation sites (Fig. S6B–D). In addition, cells overexpressing R1441C LRRK2 and 4xSA LRRK2 phosphomutant have similarly impeded autophagy in both basal and starvation conditions (Fig. 7A, B). LRRK2 pathological mutations were previously shown to impair autophagy induction during starvation in studies performed on fibroblasts obtained from patients carrying the G2019S, R1441C/G/H or Y1699C LRRK2 variants [73]. Interestingly, these effects on starvation-induced autophagy mediated by pathological LRRK2 mutants were mTORC1-independent. The disconnect between LRRK2 and mTORC1 signaling may also be manifest in the differences observed by us with regard to LRRK2 phosphorylation in starvation conditions versus treatment with the mTORC1/2 inhibitor Torin-1 (Fig. 1B–E and Fig. S1C, D). Indeed, starvation activates several signal transduction pathways leading to autophagy [74], while Torin-1 specifically inhibits mTOR [75]. We can therefore speculate that the phosphorylation of serines located in LRRK2’s phosphorylation cluster (S910/S935/S955/S973), may be downstream of one or more of other pathways activated during starvation, such as the activation of phosphatidylinositol-3-kinase or of c-Jun NH2-terminal kinase-1.

In conclusion, the retardation of autophagy observed in 4xSA LRRK2 as well as in pathological LRRK2 variants highlights the connection between the phosphorylation status of the LRRK2 constitutive sites and the kinase function of LRRK2 in both autophagy regulation and in their contribution to PD pathology. Future work will have to determine the mechanisms by which Rab8a and Rab10 interfere with the autophagic process, and identify whether other LRRK2-interacting proteins are thereby involved.

## Materials and methods

### Cell lines

Human neuroblastoma SH-SY5Y^LRRK2^ and SH-SY5Y^EV^ were generated from the CRL-2266 cell line (ATCC) by lentiviral vector transduction as described [23, 38, 41, 76]. The SH-SY5Y^4xSA LRRK2^ and the SH-SY5Y^R1441C LRRK2^ mutants were generated by the Leuven viral vector core (https://gbiomed.kuleuven.be/english/corefacilities/LVVC/) using gBlock® Gene Fragments (IDT, Leuven, Belgium) and lentiviral vectors encoding full-length LRRK2 under the control of the cytomegalovirus promoter and co-expressing a hygromycin resistance marker. Cells expressing a hygromycin resistance marker alone were included as a negative control. These cell lines are all polyclonal, being continually cultured under antibiotic selection and not having been subcloned [38]. Moreover, all cells expressed LRRK2 at similar levels (Fig. S2A, B).

MEFs were cultured as described [77]. MEF^KO LRRK2^ cells were a kind gift from Dr. Huaibin Cai (National Institute on Aging, MD, USA) [51]. These cells were used to obtain MEF cell lines stably expressing either WT human LRRK2 (MEF^hLRRK2^) or 4xSA LRRK2 (MEF^4xSA hLRRK2^) on a null background or were transduced with an empty vector (MEF^EV^). These lines were generated by lentiviral vector transduction, in an identical way as described above for the SH-SY5Y cells and are thus also polyclonal. MEF^R1441C LRRK2^ obtained from an R1441C KI mouse, and the matching WT MEFs were a kind gift from Prof. Dario Alessi (MRC Protein Phosphorylation and Ubiquitylation Unit, Univ. Dundee, UK) [78].

All cells were regularly tested for mycoplasma contamination and found negative.

### Reagents, plasmids, and enzymatic assays

Hank’s Balanced Salt Solution (Gibco^TM^/Thermo Fisher Scientific, Merelbeke, Belgium, #24020133) was used as a starvation medium. Following reagents were used: chloroquine (Sigma-Aldrich/Merck, Overijse, Belgium, #C6628), MLi-2 (Tocris/Bio-Techne, Abingdon, UK, #5756), Torin-1 (Abcam, Cambridge, UK, 218606), PF-06447475 (Axon Medchem, Groningen, Netherlands, #2546), E64d (Cayman Chemicals/Sanbio, Uden, Netherlands, #13533), and pepstatin A (Cayman Chemicals, #9000469). The pMRX-IP-GFP-LC3-RFP-LC3ΔG plasmid was obtained from Prof. Noboru Mizushima (Univ. Tokyo, Japan) via Addgene (#84572).

Lysosomal β-hexosaminidase activity was measured as described [79].

### SDS-PAGE and western blotting

Protein extraction was performed as described [77]. Samples containing an equal amount of protein were mixed with 6x Laemmli loading buffer, heated, separated by SDS-PAGE, and transferred to PVDF membranes. After blocking in 5% skimmed milk in TBST (137 mM NaCl, 2.7 mM KCl, 19 mM Tris base, 0.1% Tween), membranes were probed overnight at 4 °C with primary antibodies: anti-LRRK2 (Abcam, #133474 and #133475), anti-LRRK2 (P-S910) (Abcam, #133449), anti-LRRK2 (P-S935) (Abcam, #133450), anti-LRRK2 (P-S955) (Abcam, #169521), anti-LRRK2 (P-S973) (Abcam, 181364), anti-LC3b (Cell Signaling Technology, Leiden, Netherlands, 2775), anti-LC3 (Novus Biologicals/Bio-Techne, Abingdon, UK, #NB100-2331), anti-LAMP1 (Developmental Studies Hybridoma Bank, Univ. Iowa, IA, USA, #H4A3), anti-Rab7 (Abcam, #50533), anti-Cat L (Santa Cruz Biotechnology, Heidelberg, Germany, #390385), anti-Cat D (Cell Signaling Technology, #2284), anti-Rab8a (Abcam, #237702), anti-Rab8A (P-T72) (Abcam, #230260), anti-Rab10 (Abcam, #237703), anti-Rab10 (P-T73) (Abcam, #230261), and anti-β Actin (Cell Signaling Technology, #4970). After washing three times in TBST, membranes were incubated for 1 h at room temperature with horseradish peroxidase (HRP)-conjugated secondary antibodies, anti-rabbit or anti-mouse HRP (Cell Signaling Technology, #7074 S and #7076 S resp.) and again washed three times in TBST. Detection of proteins was obtained using Clarity Western Enhanced Chemiluminescence Substrate (Bio-Rad Laboratories, Hercules, CA, USA, #70-5061). The original western blots are made available in a Supplementary file.

### Confocal imaging

WIPI2 puncta imaging was performed as previously described [79]. The anti-WIPI2 antibody (Bio-Rad Laboratories Ltd, #MCA5780GA) was used. Observations were made with a Zeiss 710 or a Zeiss LSM880-Airyscan confocal microscope (Carl Zeiss AG, Jena, Germany) under a 63x objective, and a series of images were collected using the Z stack function. The number of WIPI2 puncta per cell was automatically quantified using CellProfiler™ software4. For LRRK2 imaging, the anti-LRRK2 antibody (Abcam, #133474) was used and the Zeiss 710 microscope. For detection, Alexa Fluor^TM^ 488-conjugated secondary antibodies (Invitrogen, **#**A-11001 and # A-11008) were used.

### Transfections with WT and phosphorylation-deficient Rab8a and Rab10

pDEST53-GFP-Rab8a, pDEST53-GFP-Rab8aT72A, pDEST53-GFP-Rab10, and pDEST53-GFP-Rab10T73A were kindly provided by Dr. Sabine Hilfiker (Spanish National Research Council, CSIC, Spain) [80]. SH-SY5Y^LRRK2^ and SH-SY5Y^4xSA LRRK2^ cells were transfected based on the previously described protocol [48]. Briefly, 80% confluent cells were transfected in six-well plates with 2 μg DNA and 6 μl Lipofectamine 2000 (Invitrogen^TM^/Thermo Fisher Scientific, Merelbeke, Belgium, #11668019) per well in 500 μl Opti-MEM (Gibco^TM^, #31985062). After 5 h, Opti-MEM was replaced with a full medium. On the next day, cells were passaged to 10 cm plates and kept for an additional 48–72 h, until reaching 70% confluence, when appropriate treatment, followed by western blot analysis, was conducted.

### Autophagic flux measurement with GFP-LC3-RFP-LC3ΔG

Cells were stably infected with pMRX-IP-GFP-LC3-RFP-LC3ΔG [45] using the Phoenix-MMULV system (https://web.stanford.edu/group/nolan/_OldWebsite/tutorials/tutorials.html) as previously used [77].

The GFP-LC3-RFP-LC3ΔG-expressing cells were cultured on 96-well, flat bottom plates, which were applied to the IncuCyte imager at 37 °C in a 5% CO_2_ incubator. Live images were acquired and analyzed using Incucyte® S3 Live-Cell Analysis System (Essen BioScience, Newark, UK). In all cases, the overall signal was captured with a 10x objective, whereby images from at least 3 wells per condition and at least two fields per well were taken. The GFP confluence/RFP confluence was determined to obtain the GFP/RFP ratio, indicative for the level of autophagic flux. Representative images of cells expressing GFP-LC3-RFP-LC3ΔG were obtained with a Zeiss 710 confocal microscope after cell culture on coverslips and fixation in 4% paraformaldehyde in PBS (15 min, room temperature).

### LysoTracker red staining

Cells were cultured on 96-well plates and stained with 50 nM LysoTracker™ Red DND-99 (Invitrogen^TM^, Thermo Fisher Scientific Ltd, L7528) for 2 h. Live images were acquired and analysed using Incucyte® S3 Live-Cell Analysis System (Essen Bioscience, Newark, UK) at 37 °C in a 5% CO_2_ incubator. The 10x objective was used and images from at least 3 wells per condition and at least 2 fields per well were taken. The confluence of the LysoTracker red-positive compartments (the percentage of the image area occupied) was determined and normalized to the total cell confluence. For representative images, cells were cultured on coverslips and fixed in 4% paraformaldehyde for 15 min at room temperature. Coverslips were washed with PBS and mounted on slides using Dako immunofluorescence mounting solution (Agilent Technologies, S3023). Images were obtained using the Zeiss 710 confocal microscope (Carl Zeiss AG) under a 63x objective.

### Statistical analyses

Statistical significance was obtained using GraphPad Prism as indicated in the legends to the figures.

## Supplementary information

Legends to supplementary figures

Supplementary figure 1

Supplementary figure 2

Supplementary figure 3

Supplementary figure 4

Supplementary figure 5

Supplementary figure 6

Kania et al - aj-checklist

Full-size blots

## Data Availability

The datasets generated during and/or analysed during the current study are available from the corresponding authors on reasonable request.

## References

[1] Lewis PA (2009). The function of ROCO proteins in health and disease. Biol Cell.

[2] Hui KY, Fernandez-Hernandez H, Hu J, Schaffner A, Pankratz N, Hsu NY (2018). Functional variants in the LRRK2 gene confer shared effects on risk for Crohn’s disease and Parkinson’s disease. Sci Transl Med.

[3] Saunders-Pullman R, Barrett MJ, Stanley KM, San Luciano M, Shanker V, Severt L (2010). LRRK2 G2019S mutations are associated with an increased cancer risk in Parkinson disease. Mov Disord.

[4] Paisán-Ruiz C, Jain S, Evans EW, Gilks WP, Simon J, van der Brug M (2004). Cloning of the gene containing mutations that cause PARK8-linked Parkinson’s disease. Neuron.

[5] Zimprich A, Biskup S, Leitner P, Lichtner P, Farrer M, Lincoln S (2004). Mutations in LRRK2 cause autosomal-dominant parkinsonism with pleomorphic pathology. Neuron.

[6] Kluss JH, Mamais A, Cookson MR (2019). LRRK2 links genetic and sporadic Parkinson’s disease. Biochem Soc Trans.

[7] Beilina A, Rudenko IN, Kaganovich A, Civiero L, Chau H, Kalia SK (2014). Unbiased screen for interactors of leucine-rich repeat kinase 2 supports a common pathway for sporadic and familial Parkinson disease. Proc Natl Acad Sci USA.

[8] Guaitoli G, Raimondi F, Gilsbach BK, Gomez-Llorente Y, Deyaert E, Renzi F (2016). Structural model of the dimeric Parkinson’s protein LRRK2 reveals a compact architecture involving distant interdomain contacts. Proc Natl Acad Sci USA.

[9] Kania E, Parys JB (2019). The emerging interrelation between ROCO and related kinases, intracellular Ca^2+^ signaling, and autophagy. Biochim Biophys Acta Mol Cell Res.

[10] Berwick DC, Heaton GR, Azeggagh S, Harvey K (2019). LRRK2 biology from structure to dysfunction: research progresses, but the themes remain the same. Mol Neurodegener.

[11] Paisan-Ruiz C, Lewis PA, Singleton AB (2013). LRRK2: cause, risk, and mechanism. J Parkinsons Dis.

[12] Mata IF, Davies MJ, Lopez AN, Dorschner MO, Martinez E, Yearout D (2017). The discovery of LRRK2 p.R1441S, a novel mutation for Parkinson’s disease, adds to the complexity of a mutational hotspot. Am J Med Genet B Neuropsychiatr Genet.

[13] Gasser T (2009). Molecular pathogenesis of Parkinson disease: insights from genetic studies. Expert Rev Mol Med.

[14] Aasly JO, Vilariño-Güell C, Dachsel JC, Webber PJ, West AB, Haugarvoll K (2010). Novel pathogenic LRRK2 p.Asn1437His substitution in familial Parkinson’s disease. Mov Disord.

[15] Thaler A, Ash E, Gan-Or Z, Orr-Urtreger A, Giladi N (2009). The LRRK2 G2019S mutation as the cause of Parkinson’s disease in Ashkenazi Jews. J Neural Transm.

[16] Guo L, Gandhi PN, Wang W, Petersen RB, Wilson-Delfosse AL, Chen SG (2007). The Parkinson’s disease-associated protein, leucine-rich repeat kinase 2 (LRRK2), is an authentic GTPase that stimulates kinase activity. Exp Cell Res.

[17] Webber PJ, Smith AD, Sen S, Renfrow MB, Mobley JA, West AB (2011). Autophosphorylation in the leucine-rich repeat kinase 2 (LRRK2) GTPase domain modifies kinase and GTP-binding activities. J Mol Biol.

[18] De Wit T, Baekelandt V, Lobbestael E (2018). LRRK2 phosphorylation: behind the scenes. Neuroscientist.

[19] Marchand A, Droyer M, Sarchione A, Chartier-Harlin MC, Taymans JM (2020). LRRK2 phosphorylation, more than an epiphenomenon. Front Neurosci.

[20] Muda K, Bertinetti D, Gesellchen F, Hermann JS, von Zweydorf F, Geerlof A (2014). Parkinson-related LRRK2 mutation R1441C/G/H impairs PKA phosphorylation of LRRK2 and disrupts its interaction with 14-3-3. Proc Natl Acad Sci USA.

[21] Li X, Wang QJ, Pan N, Lee S, Zhao Y, Chait BT (2011). Phosphorylation-dependent 14-3-3 binding to LRRK2 is impaired by common mutations of familial Parkinson’s disease. PLoS ONE.

[22] Chia R, Haddock S, Beilina A, Rudenko IN, Mamais A, Kaganovich A (2014). Phosphorylation of LRRK2 by casein kinase 1α regulates trans-Golgi clustering via differential interaction with ARHGEF7. Nat Commun.

[23] De Wit T, Baekelandt V, Lobbestael E (2019). Inhibition of LRRK2 or casein kinase 1 results in LRRK2 protein destabilization. Mol Neurobiol.

[24] Dzamko N, Inesta-Vaquera F, Zhang J, Xie C, Cai H, Arthur S (2012). The IkappaB kinase family phosphorylates the Parkinson’s disease kinase LRRK2 at Ser935 and Ser910 during Toll-like receptor signaling. PLoS ONE.

[25] Padmanabhan S, Lanz TA, Gorman D, Wolf M, Joyce A, Cabrera C (2020). An assessment of LRRK2 serine 935 phosphorylation in human peripheral blood mononuclear cells in idiopathic Parkinson’s disease and G2019S LRRK2 cohorts. J Parkinsons Dis.

[26] Iannotta L, Biosa A, Kluss JH, Tombesi G, Kaganovich A, Cogo S (2020). Divergent effects of G2019S and R1441C LRRK2 mutations on LRRK2 and Rab10 phosphorylations in mouse tissues. Cells.

[27] Fan Y, Nirujogi RS, Garrido A, Ruiz-Martínez J, Bergareche-Yarza A, Mondragón-Rezola E (2021). R1441G but not G2019S mutation enhances LRRK2 mediated Rab10 phosphorylation in human peripheral blood neutrophils. Acta Neuropathol.

[28] Kalogeropulou AF, Purlyte E, Tonelli F, Lange SM, Wightman M, Prescott AR (2022). Impact of 100 LRRK2 variants linked to Parkinson’s disease on kinase activity and microtubule binding. Biochem J.

[29] Tong Y, Giaime E, Yamaguchi H, Ichimura T, Liu Y, Si H (2012). Loss of leucine-rich repeat kinase 2 causes age-dependent bi-phasic alterations of the autophagy pathway. Mol Neurodegener.

[30] Ho DH, Kim H, Nam D, Sim H, Kim J, Kim HG (2018). LRRK2 impairs autophagy by mediating phosphorylation of leucyl-tRNA synthetase. Cell Biochem Funct.

[31] Sánchez-Danés A, Richaud-Patin Y, Carballo-Carbajal I, Jiménez-Delgado S, Caig C, Mora S (2012). Disease-specific phenotypes in dopamine neurons from human iPS-based models of genetic and sporadic Parkinson’s disease. EMBO Mol Med.

[32] Plowey ED, Cherra SJ, Liu YJ, Chu CT (2008). Role of autophagy in G2019S-LRRK2-associated neurite shortening in differentiated SH-SY5Y cells. J Neurochem.

[33] Wallings R, Connor-Robson N, Wade-Martins R (2019). LRRK2 interacts with the vacuolar-type H^+^-ATPase pump a1 subunit to regulate lysosomal function. Hum Mol Genet.

[34] Alegre-Abarrategui J, Christian H, Lufino MMP, Mutihac R, Lourenço Venda L, Ansorge O (2009). LRRK2 regulates autophagic activity and localizes to specific membrane microdomains in a novel human genomic reporter cellular model. Hum Mol Genet.

[35] Zhao Y, Keshiya S, Atashrazm F, Gao J, Ittner LM, Alessi D (2018). Nigrostriatal pathology with reduced astrocytes in LRRK2 S910/S935 phosphorylation deficient knockin mice. Neurobiol Dis.

[36] West AB, Moore DJ, Choi C, Andrabi SA, Li X, Dikeman D (2007). Parkinson’s disease-associated mutations in LRRK2 link enhanced GTP-binding and kinase activities to neuronal toxicity. Hum Mol Genet.

[37] Pellegrini L, Hauser DN, Li Y, Mamais A, Beilina A, Kumaran R (2018). Proteomic analysis reveals co-ordinated alterations in protein synthesis and degradation pathways in LRRK2 knockout mice. Hum Mol Genet.

[38] Lobbestael E, Civiero L, De Wit T, Taymans JM, Greggio E, Baekelandt V (2016). Pharmacological LRRK2 kinase inhibition induces LRRK2 protein destabilization and proteasomal degradation. Sci Rep.

[39] Obergasteiger J, Frapporti G, Lamonaca G, Pizzi S, Picard A, Lavdas A (2020). Kinase inhibition of G2019S-LRRK2 enhances autolysosome formation and function to reduce endogenous alpha-synuclein intracellular inclusions. Cell Death Discov.

[40] Dooley HC, Razi M, Polson HEJ, Girardin SE, Wilson MI, Tooze SA (2014). WIPI2 links LC3 conjugation with PI3P, autophagosome formation, and pathogen clearance by recruiting Atg12-5-16L1. Mol Cell.

[41] Vancraenenbroeck R, De Raeymaecker J, Lobbestael E, Gao F, De Maeyer M, Voet A (2014). In silico, in vitro and cellular analysis with a kinome-wide inhibitor panel correlates cellular LRRK2 dephosphorylation to inhibitor activity on LRRK2. Front Mol Neurosci.

[42] Purlyte E, Dhekne HS, Sarhan AR, Gomez R, Lis P, Wightman M (2018). Rab29 activation of the Parkinson’s disease-associated LRRK2 kinase. EMBO J.

[43] Fell MJ, Mirescu C, Basu K, Cheewatrakoolpong B, DeMong DE, Ellis JM (2015). MLi-2, a potent, selective, and centrally active compound for exploring the therapeutic potential and safety of LRRK2 kinase inhibition. J Pharmacol Exp Ther.

[44] Henderson JL, Kormos BL, Hayward MM, Coffman KJ, Jasti J, Kurumbail RG, et al. Discovery and preclinical profiling of 3-[4-(morpholin-4-yl)-7H-pyrrolo[2,3-d]pyrimidin-5-yl]benzonitrile (PF-06447475), a highly potent, selective, brain penetrant, and in vivo active LRRK2 kinase inhibitor. J Med Chem. 2015;58:419–32. 10.1021/jm5014055 25353650

[45] Kaizuka T, Morishita H, Hama Y, Tsukamoto S, Matsui T, Toyota Y (2016). An autophagic flux probe that releases an internal control. Mol Cell.

[46] De Groot PG, Ovde Elferink RO, Hollemans M, Strijland A, Westerveld A, Meera Khan P (1981). Inactivation by chloroquine of alpha-galactosidase in cultured human skin fibroblasts. Exp Cell Res.

[47] Eguchi T, Kuwahara T, Sakurai M, Komori T, Fujimoto T, Ito G (2018). LRRK2 and its substrate Rab GTPases are sequentially targeted onto stressed lysosomes and maintain their homeostasis. Proc Natl Acad Sci USA.

[48] Madero-Pérez J, Fdez E, Fernández B, Lara Ordónez AJ, Blanca Ramírez M, Gómez-Suaga P (2018). Parkinson disease-associated mutations in LRRK2 cause centrosomal defects via Rab8a phosphorylation. Mol Neurodegener.

[49] Steger M, Tonelli F, Ito G, Davies P, Trost M, Vetter M (2016). Phosphoproteomics reveals that Parkinson’s disease kinase LRRK2 regulates a subset of Rab GTPases. Elife.

[50] Kluss JH, Conti MM, Kaganovich A, Beilina A, Melrose HL, Cookson MR (2018). Detection of endogenous S1292 LRRK2 autophosphorylation in mouse tissue as a readout for kinase activity. NPJ Parkinsons Dis.

[51] Lin X, Parisiadou L, Gu XL, Wang L, Shim H, Sun L (2009). Leucine-rich repeat kinase 2 regulates the progression of neuropathology induced by Parkinson’s-disease-related mutant α-synuclein. Neuron.

[52] Cho HJ, Xie C, Cai H (2018). AGE-induced neuronal cell death is enhanced in G2019S LRRK2 mutation with increased RAGE expression. Transl Neurodegener.

[53] Iaccarino C, Crosio C, Vitale C, Sanna G, Carri MT, Barone P (2007). Apoptotic mechanisms in mutant LRRK2-mediated cell death. Hum Mol Genet.

[54] Jiang ZC, Chen XJ, Zhou Q, Gong XH, Chen X, Wu WJ (2019). Downregulated LRRK2 gene expression inhibits proliferation and migration while promoting the apoptosis of thyroid cancer cells by inhibiting activation of the JNK signaling pathway. Int J Oncol.

[55] Chen Z, Cao Z, Zhang W, Gu M, Zhou ZD, Li B (2017). LRRK2 interacts with ATM and regulates Mdm2-p53 cell proliferation axis in response to genotoxic stress. Hum Mol Genet.

[56] Wallings R, Manzoni C, Bandopadhyay R (2015). Cellular processes associated with LRRK2 function and dysfunction. FEBS J.

[57] Cookson MR (2016). Cellular functions of LRRK2 implicate vesicular trafficking pathways in Parkinson’s disease. Biochem Soc Trans.

[58] Stormo AED, Shavarebi F, FitzGibbon M, Earley EM, Ahrendt H, Lum LS (2022). The E3 ligase TRIM1 ubiquitinates LRRK2 and controls its localization, degradation, and toxicity. J Cell Biol.

[59] Schapansky J, Nardozzi JD, Felizia F, LaVoie MJ (2014). Membrane recruitment of endogenous LRRK2 precedes its potent regulation of autophagy. Hum Mol Genet.

[60] Mamais A, Chia R, Beilina A, Hauser DN, Hall C, Lewis PA (2014). Arsenite stress down-regulates phosphorylation and 14-3-3 binding of leucine-rich repeat kinase 2 (LRRK2), promoting self-association and cellular redistribution. J Biol Chem.

[61] Marchand A, Sarchione A, Athanasopoulos PS, Bauderlique-Le Roy H, Goveas L, Magnez R (2022). A phosphosite mutant approach on LRRK2 links phosphorylation and dephosphorylation to protective and deleterious markers, respectively. Cells.

[62] Liu Y, Gray NS (2006). Rational design of inhibitors that bind to inactive kinase conformations. Nat Chem Biol.

[63] Tasegian A, Singh F, Ganley IG, Reiyh AD, Alessi DR (2021). Impact of Type II LRRK2 inhibitors on signaling and mitophagy. Biochem J.

[64] Madureira M, Connor-Robson N, Wade-Martins R (2020). LRRK2: autophagy and lysosomal activity. Front Neurosci.

[65] Sanyal A, DeAndrade MP, Novis HS, Lin S, Chang J, Lengacher N (2020). Lysosome and inflammatory defects in GBA1-mutant astrocytes are normalized by LRRK2 inhibition. Mov Disord.

[66] Kuwahara T, Funakawa K, Komori T, Sakurai M, Yoshii G, Eguchi T (2020). Roles of lysosomotropic agents on LRRK2 activation and Rab10 phosphorylation. Neurobiol Dis.

[67] Herbst S, Campbell P, Harvey J, Bernard EM, Papayannopoulos V, Wood NW (2020). LRRK2 activation controls the repair of damaged endomembranes in macrophages. EMBO J.

[68] Bonet-Ponce L, Beilina A, Williamson CD, Lindberg E, Kluss JH, Saez-Atienzar S (2020). LRRK2 mediates tubulation and vesicle sorting from lysosomes. Sci Adv.

[69] Ito G, Katsemonova K, Tonelli F, Lis P, Baptista MAS, Shpiro N (2016). Phos-tag analysis of Rab10 phosphorylation by LRRK2: a powerful assay for assessing kinase function and inhibitors. Biochem J.

[70] Dzamko N, Gysbers AM, Bandopadhyay R, Bolliger MF, Uchino A, Zhao Y (2017). LRRK2 levels and phosphorylation in Parkinson’s disease brain and cases with restricted Lewy bodies. Mov Disord.

[71] Ohta E, Kubo M, Obata F (2010). Prevention of intracellular degradation of I2020T mutant LRRK2 restores its protectivity against apoptosis. Biochem Biophys Res Commun.

[72] Greene ID, Mastaglia F, Meloni BP, West KA, Chieng J, Mitchell CJ (2014). Evidence that the LRRK2 ROC domain Parkinson’s disease-associated mutants A1442P and R1441C exhibit increased intracellular degradation. J Neurosci Res.

[73] Manzoni C, Mamais A, Dihanich S, McGoldrick P, Devine MJ, Zerle J (2013). Pathogenic Parkinson’s disease mutations across the functional domains of LRRK2 alter the autophagic/lysosomal response to starvation. Biochem Biophys Res Commun.

[74] Ravikumar B, Sarkar S, Davies JE, Futter M, Garcia-Arencibia M, Green-Thompson ZW (2010). Regulation of mammalian autophagy in physiology and pathophysiology. Physiol Rev.

[75] Liu Q, Chang JW, Wang J, Kang SA, Thoreen CC, Markhard A (2010). Discovery of 1-(4-(4-propionylpiperazin-1-yl)-3-(trifluoromethyl)phenyl)-9-(quinolin-3-yl)benz o[h][1,6]naphthyridin-2(1H)-one as a highly potent, selective mammalian target of rapamycin (mTOR) inhibitor for the treatment of cancer. J Med Chem.

[76] Daniels V, Vancraenenbroeck R, Law BMH, Greggio E, Lobbestael E, Gao F (2011). Insight into the mode of action of the LRRK2 Y1699C pathogenic mutant. J Neurochem.

[77] Beaumatin F, O’Prey J, Barthet VJA, Zunino B, Parvy JP, Bachmann AM (2019). mTORC1 activation requires DRAM-1 by facilitating lysosomal amino acid efflux. Mol Cell.

[78] Kalogeropulou AF, Freemantle JB, Lis P, Vides EG, Polinski NK, Alessi DR (2020). Endogenous Rab29 does not impact basal or stimulated LRRK2 pathway activity. Biochem J.

[79] Sakamaki JI, Wilkinson S, Hahn M, Tasdemir N, O’Prey J, Clark W (2017). Bromodomain Protein BRD4 is a transcriptional repressor of autophagy and lysosomal function. Mol Cell.

[80] Lara Ordónez AJ, Fernández B, Fdez E, Romo-Lozano M, Madero-Pérez J, Lobbestael E (2019). RAB8, RAB10 and RILPL1 contribute to both LRRK2 kinase-mediated centrosomal cohesion and ciliogenesis deficits. Hum Mol Genet.

