# Supplementary material for: LRRK2 phosphorylation status and kinase activity regulate (macro)autophagy in a Rab8a/Rab10-dependent manner
Source: Cell Death Dis. 2023 Jul 15;14(7):436. doi: 10.1038/s41419-023-05964-0 (PMC10349885; doi:10.1038/s41419-023-05964-0)
Supplement: Supplementary file 1 — Legends to supplementary figures [file 41419_2023_5964_MOESM1_ESM.docx]

**Legends to the Supplementary figures**

**Figure S1.** Increased LRRK2 phosphorylation in starvation-treated MEF^hLRRK2^ cells. (**A**) Western blots showing total LRRK2 levels detected with two different antibodies (ab133474 and ab133475, Abcam) in MEF^KO LRRK2^ (KO) or MEF^hLRRK2^ (WT) cells. β actin was used as a loading control. (**B-D**) MEF^hLRRK2^ cells were treated for 2 h with DMSO (CTRL), starvation medium (Starv.) or Torin 1 (50 nM). Representative western blots showing (**B**) LC3 I and II levels (N=3 independent experiments) and (**C**) total LRRK2 expression levels as well as its phosphorylation at S935/S910/S955/S973 (N=4-5 independent experiments). (**D**) Quantification of LRRK2 expression and phosphorylation levels at S935/S910/S955/S973 presented in (**C**). β actin was used on all blots as a loading control. All graphs are presented as plots of individual data points with mean ± SD with each experiment marked in a different color and normalized to control condition (no starvation medium). Statistical analysis was performed using a Repeated Measures one-way ANOVA with Dunnett’s multiple comparisons post-test (*P<0.05;**P<0.001).

**Figure S2.** Cells expressing the 4xSA LRRK2 phospho-dead mutant do not demonstrate LRRK2 phosphorylation. (**A**) Western blots showing LRRK2 expression and phosphorylation levels at S910/S935/S955/S973 in SH-SY5Y^EV^ (EV), SH-SY5Y^LRRK2^ (WT), and SH-SY5Y^4xSA LRRK2^ (4xSA) cells. **(B)** Representative images showing LRRK2 staining (green) and nuclei stained with DAPI (blue) in untreated SH-SY5Y^LRRK2^ and SH-SY5Y^4xSA LRRK2^ cells. Scale bar = 20 μm. (**C**) Western blots showing LRRK2 expression and phosphorylation levels at S910/S935/S955/S973 in MEF^EV^ (EV), MEF^hLRRK2^ (WT), and MEF^4xSA hLRRK2^ (4xSA) cells. For both (**A**) and (**B**) β actin was used as loading control.

**Figure S3.** Starvation-induced autophagy is impaired in cells expressing the 4xSA LRRK2 phospho-dead mutant. (**A**-**B**) MEF^EV^ (EV), MEF^hLRRK2^ (WT), and MEF^4xSA hLRRK2^ (4xSA) cells remained untreated (CTRL) or were treated for 2 h with starvation medium (Starv.). (**A**) Representative images showing WIPI2 puncta from N=6 independent experiments with at least 800 cells analyzed per condition. Scale bar: 20 μm. (**B**) Quantification of the WIPI2 puncta per cell shown in (**A**). The means of all experiments ± SEM are shown with each experiment marked in a different color. For statistical analysis Repeated Measures two-way ANOVA with Sidak’s multiple comparisons post-test (*P<0.05) was used. (**C**) SH-SY5Y^EV^ (EV), SH-SY5Y^LRRK2^ (WT), and SH-SY5Y^4xSA LRRK2^ (4xSA) cells were pretreated for 4 h without or with the lysosomal degradation inhibitor chloroquine (CQ, 10 μM), as indicated. Subsequently, cells remained untreated or were treated for 2 h with starvation medium (Starv.) still without or with CQ (10 μM). Representative western blot showing LC3 I and II levels (N=5 independent experiments). β actin was used as loading control.

**Figure S4.** Decreased LRRK2 phosphorylation at S910/S935/S955/S973 in the presence of LRRK2 kinase inhibitors. SH-SY5Y^LRRK2^ cells were treated for 2 h with DMSO or with LRRK2 kinase inhibitors MLi-2 (100 nM) or PF-06447475 (150 nM). Representative western blots (N=3 independent experiments) showing LRRK2 expression levels and its phosphorylation at S910/S935/S955/S973. β actin was used as loading control.

**Figure S5**. SH-SY5Y^LRRK2^ and SH-SY5Y^4xSA LRRK2^ cells have similar numbers of lysosomes and their acidification. (**A**-**B**) SH-SY5Y^LRRK2^ and SH-SY5Y^4xSA LRRK2^ cells were treated for 4 h with DMSO (CTRL), starvation medium (Starv.) or Bafilomycin A1 (Baf A1; 200 nM) and were stained for the last 2 h of the experiment with LysoTracker Red (50 nM). (**A**) Representative images showing LysoTracker Red staining; scale bar = 20 μm. (**B**) Quantification of the area of LysoTracker Red staining for a total of N= 5 independent experiments performed, each with 3-6 technical replicates. The graph is presented as a plot of individual data points with mean ± SD, with each experiment marked in a different color. Statistical analysis was performed using a Repeated Measures two-way ANOVA with Sidak’s multiple comparisons post-test (**P<0.01; ***P<0.001).

**Figure S6.** LRRK2 kinase activity and LRRK2 phosphorylation at S910/S935/S955/S973 in cells expressing R1441C LRRK2. (**A**) SH-SY5Y^LRRK2^ (WT), SH-SY5Y^4xSA LRRK2^ (4xSA) and SH-SY5Y^R1441C LRRK2^ (RC) cells were treated for 2 h with DMSO or the LRRK2 kinase inhibitor MLi-2 (100 nM). Representative western blots showing the levels of total Rab8a, total Rab10 and the phosphorylation levels of Rab8a at T72 and of Rab10 at T73 (N=4 independent experiments). β actin was used as loading control. (**B**) SH-SY5Y^LRRK2^ (WT), SH-SY5Y^4xSA LRRK2^ (4xSA) and SH-SY5Y^R1441C LRRK2^ (RC) cells were treated for 2 h with DMSO or with starvation medium (Starv.) in the absence or presence of MLi-2 (100 nM). Representative western blots showing LRRK2 expression and its phosphorylation levels at S910/S935/S955/S973 (N=3-4 independent experiments). β actin was used as loading control. (**C**) Quantification of LRRK2 phosphorylation levels at S910/S935/S955/S973 presented in (**B**), relative to total LRRK2 levels shown as a mean ± SD and normalized to the control condition (SH-SY5Y^LRRK2^, no starvation, no MLi-2). Statistical significance is shown between SH-SY5Y^LRRK2^ and SH-SY5Y^R1441C LRRK2^ cells, both in the presence or absence of starvation; statistical analysis was performed using a one sample t-test (comparison in control conditions) or a paired, one-tailed t-test (comparison in starvation conditions) (*P<0.05;**P<0.01; ***P<0.001). (**D**) WT MEFs or MEF^R1441C LRRK2^ cells were treated with starvation medium (Starv.) for 2 h, as indicated. LRRK2 expression levels and its phosphorylation at S935 was examined. Representative western blots and the quantification from N=3 independent experiments is shown. Statistical analysis was performed using a Repeated Measures two-way ANOVA with Sidak’s multiple comparisons post-test (*P<0.05).
