# Supplementary figures and images for: LRRK2 phosphorylation status and kinase activity regulate (macro)autophagy in a Rab8a/Rab10-dependent manner
Source: Cell Death Dis. 2023 Jul 15;14(7):436. doi: 10.1038/s41419-023-05964-0 (PMC10349885; doi:10.1038/s41419-023-05964-0)

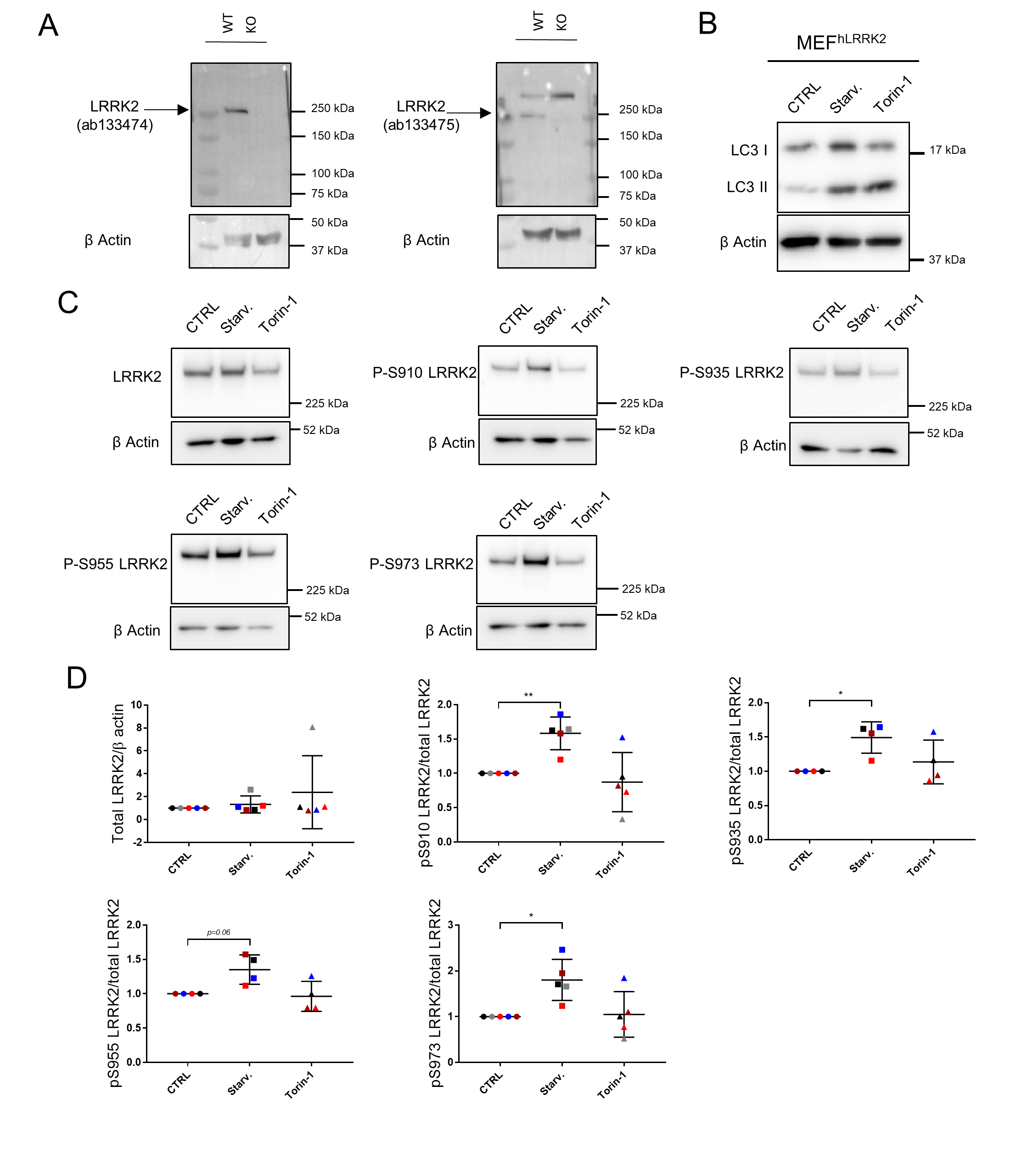

Supplement: Supplementary file 2 — Supplementary figure 1 [file 41419_2023_5964_MOESM2_ESM.tif]

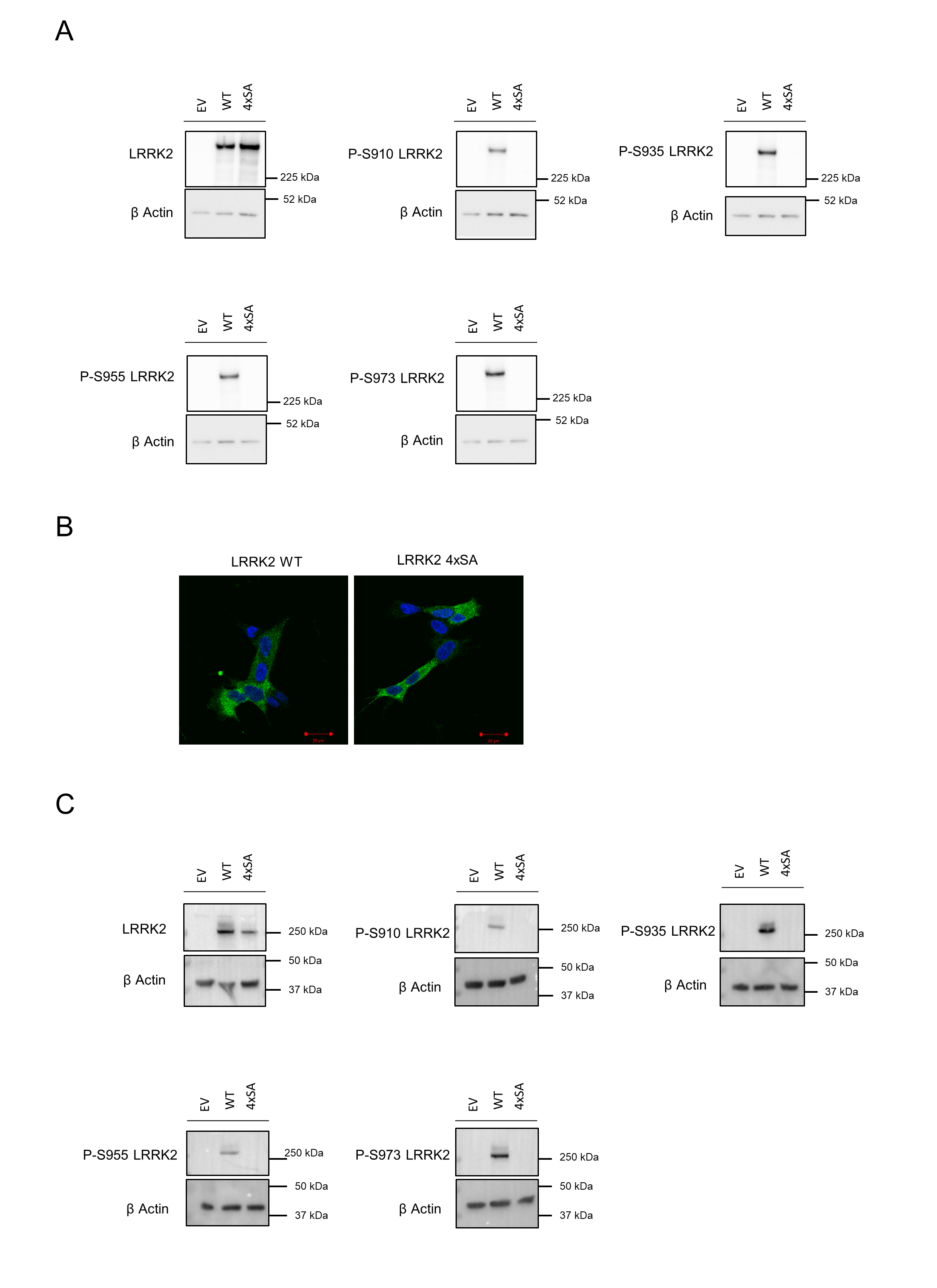

Supplement: Supplementary file 3 — Supplementary figure 2 [file 41419_2023_5964_MOESM3_ESM.tif]

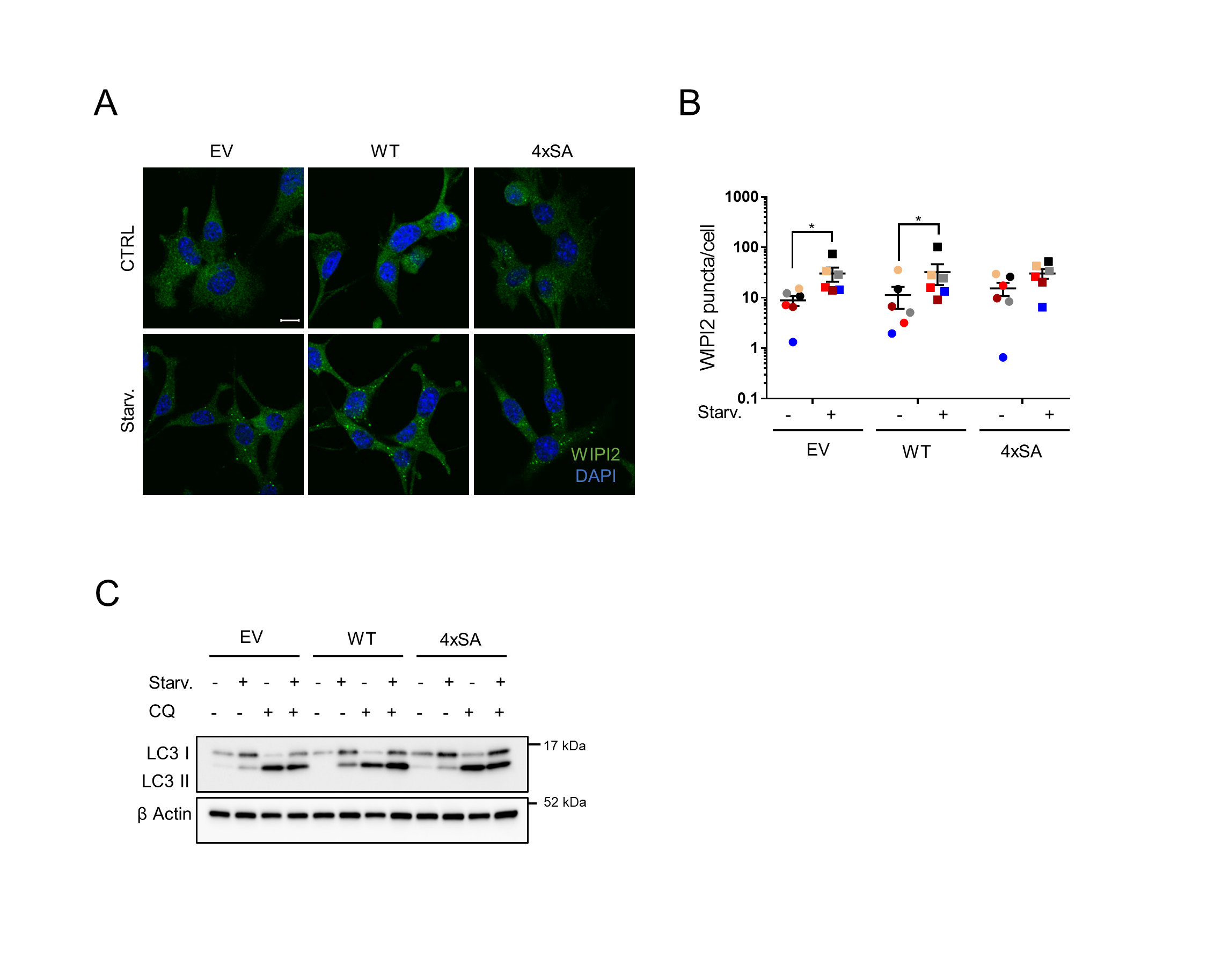

Supplement: Supplementary file 4 — Supplementary figure 3 [file 41419_2023_5964_MOESM4_ESM.tif]

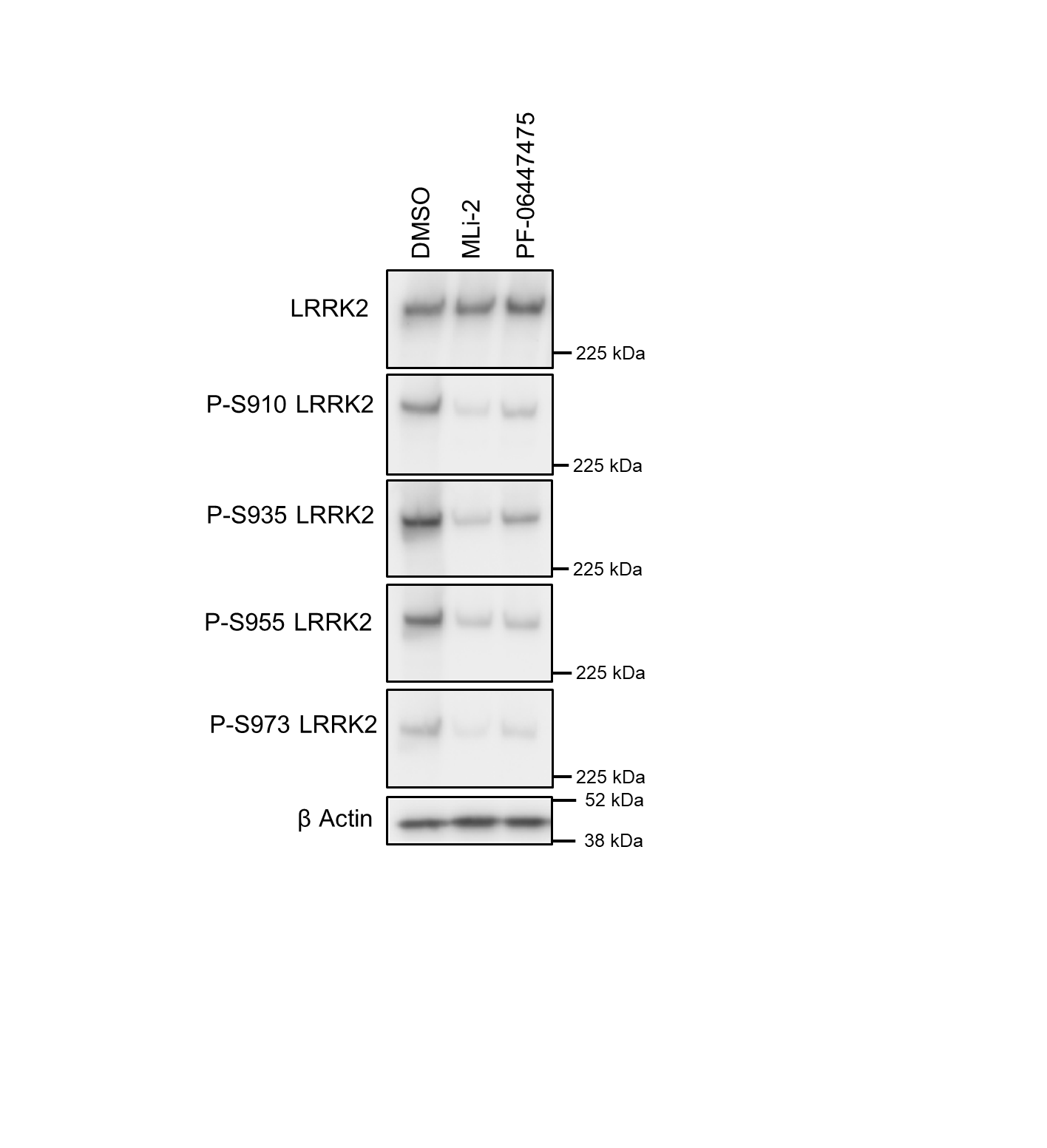

Supplement: Supplementary file 5 — Supplementary figure 4 [file 41419_2023_5964_MOESM5_ESM.tif]

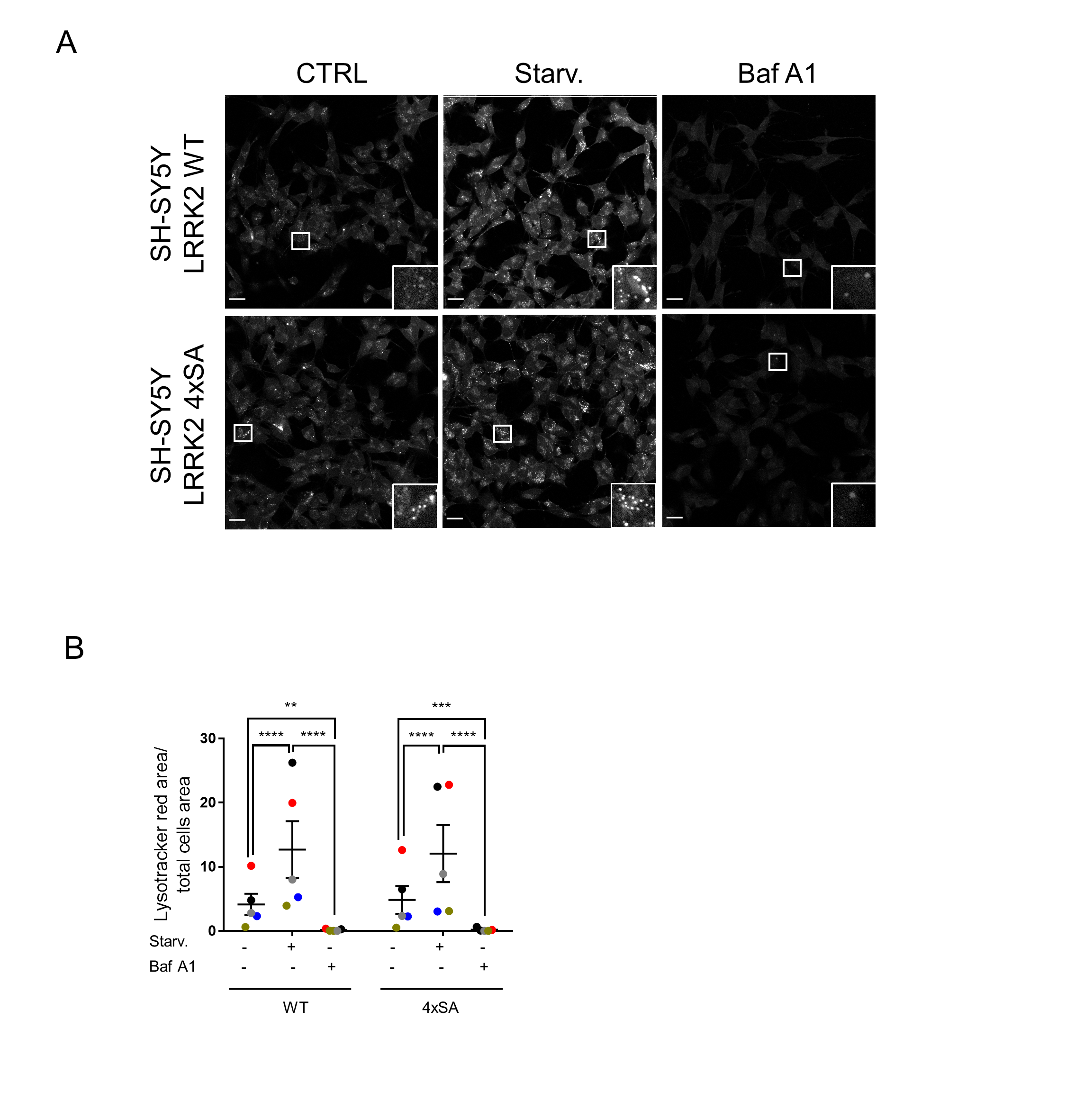

Supplement: Supplementary file 6 — Supplementary figure 5 [file 41419_2023_5964_MOESM6_ESM.tif]

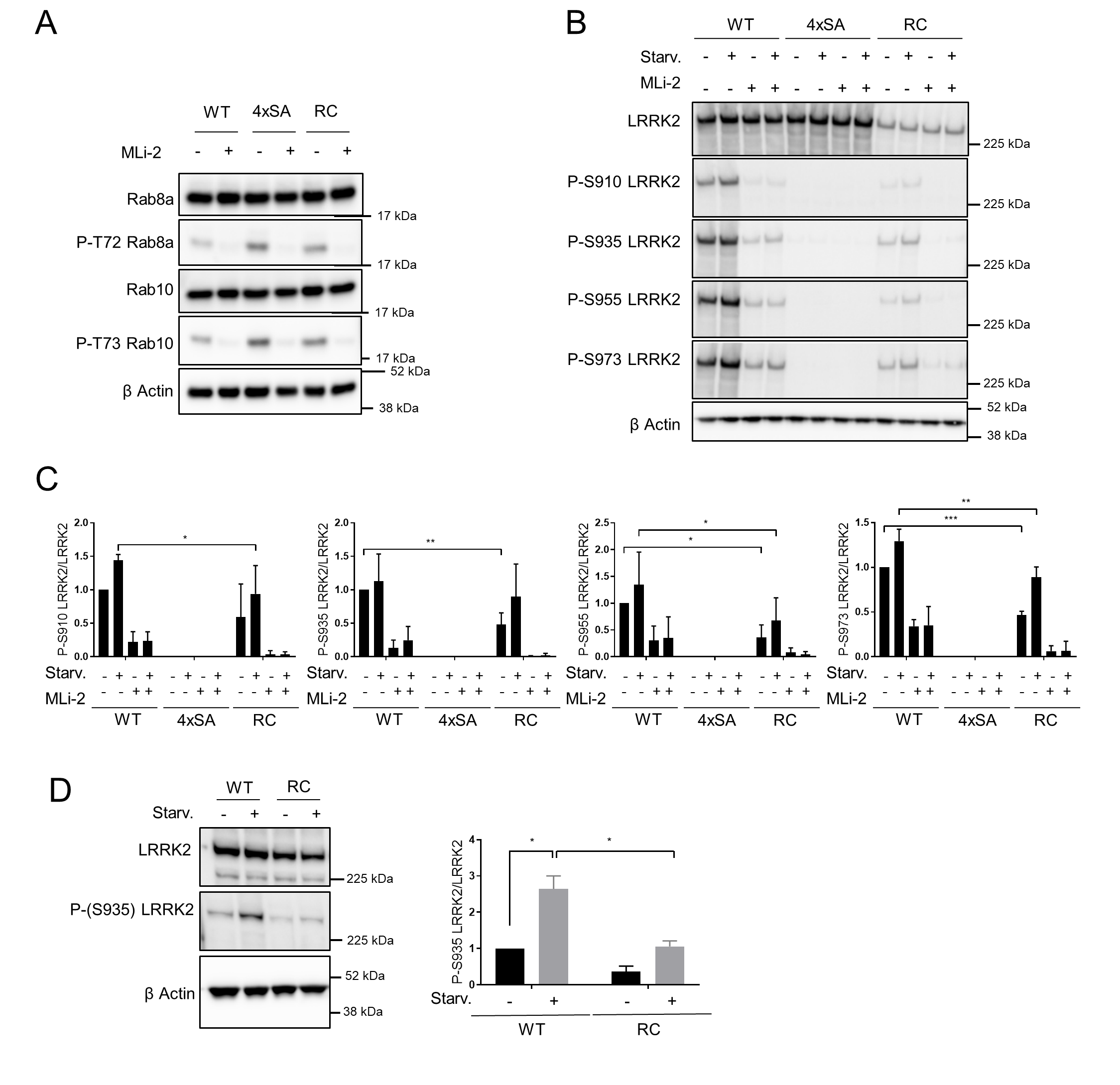

Supplement: Supplementary file 7 — Supplementary figure 6 [file 41419_2023_5964_MOESM7_ESM.tif]

Fig.1

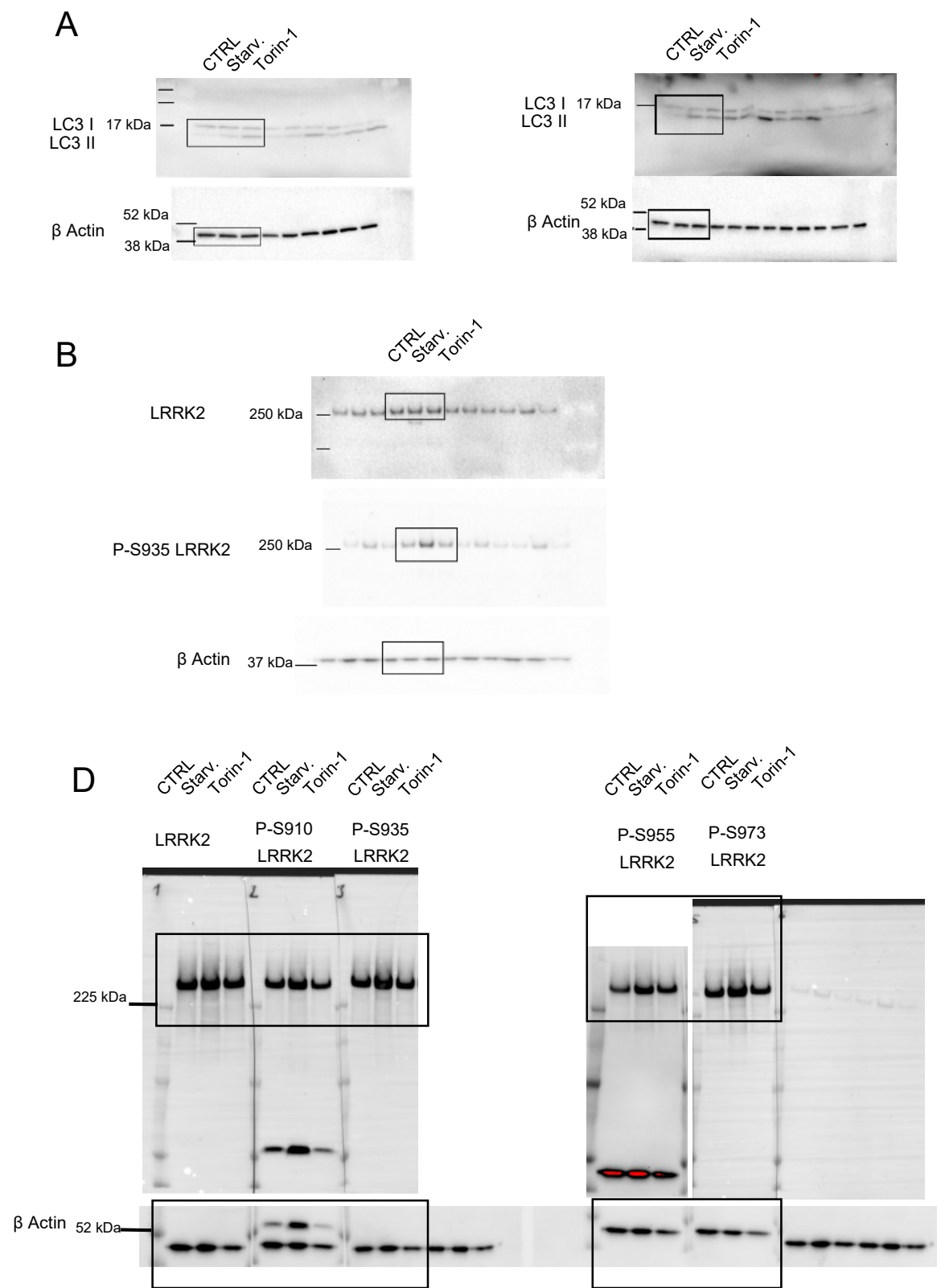

Fig.2

C

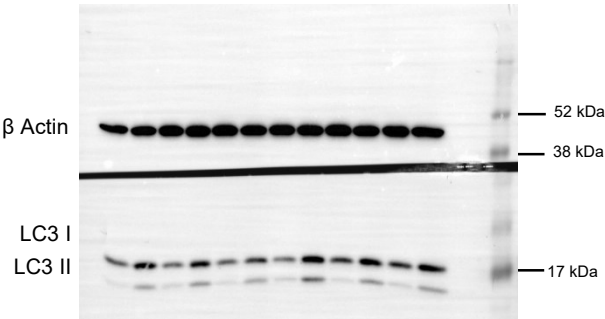

E

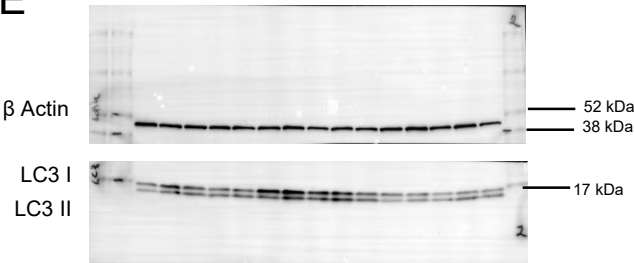

Fig.4

A

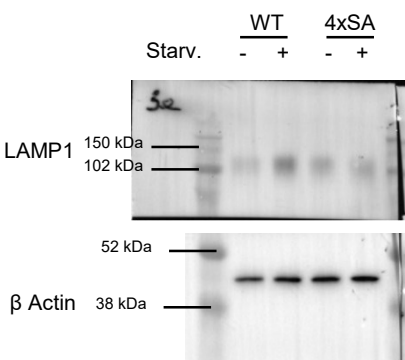

B

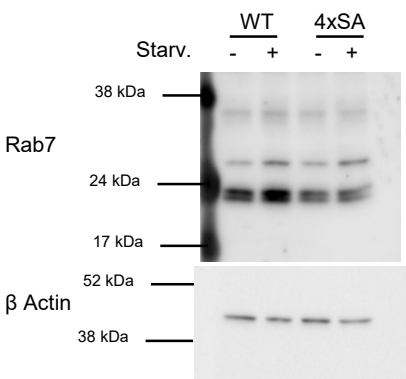

C

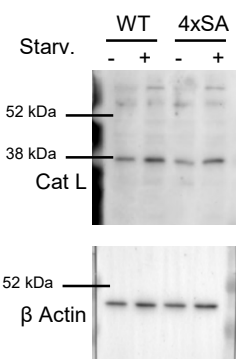

D

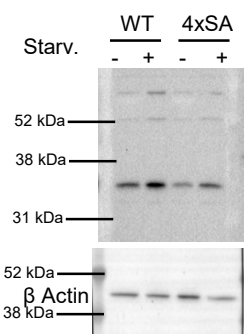

Fig. 5

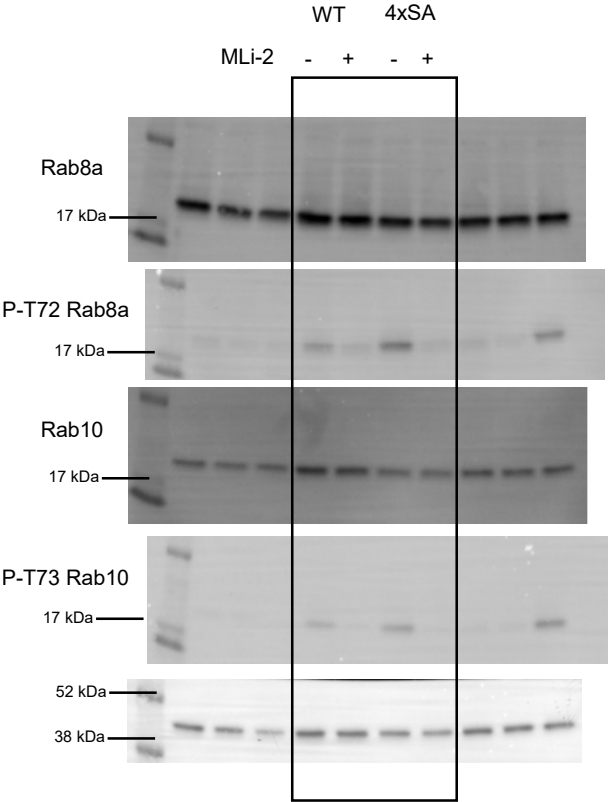

Fig. 6

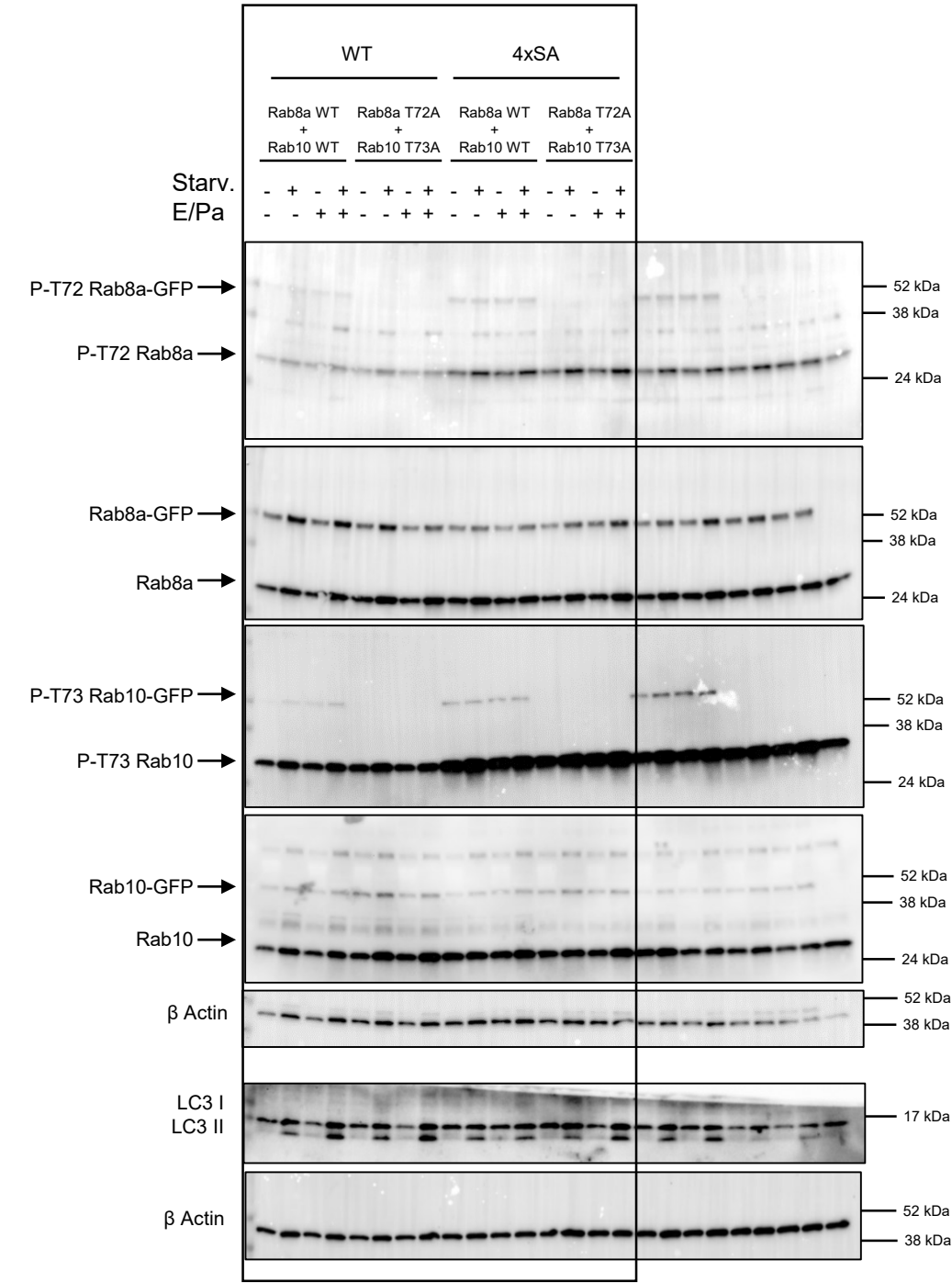

# Supplementary figures

A

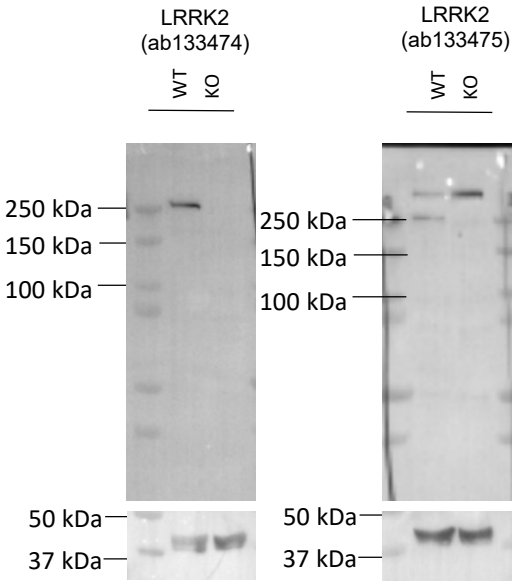

B

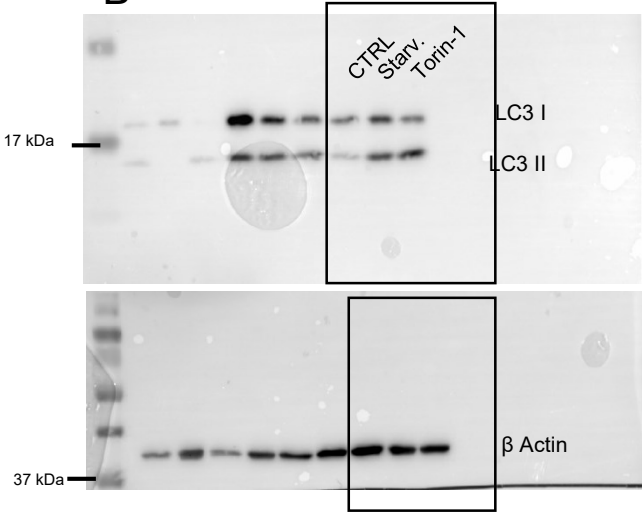

C

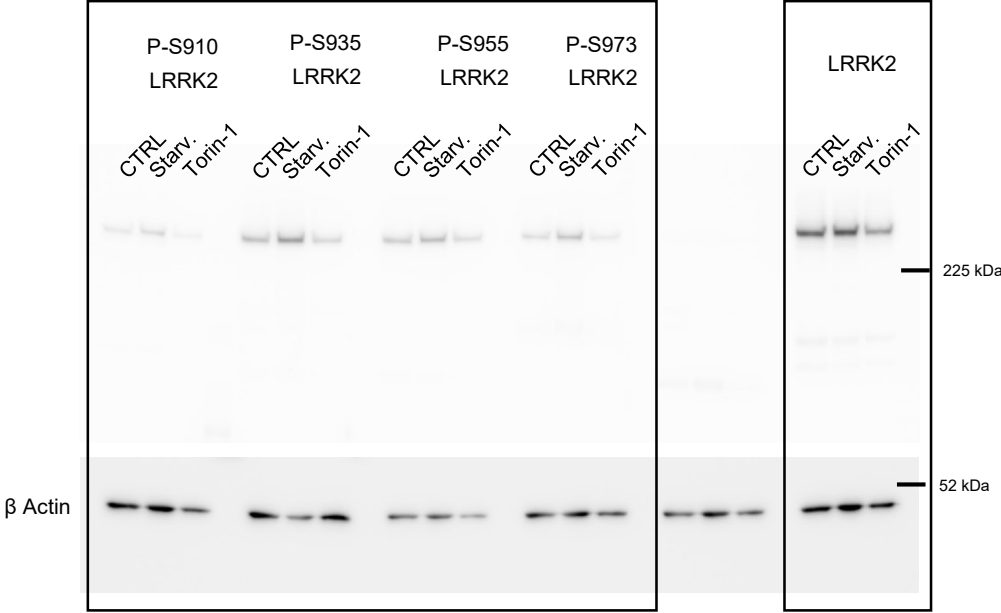

A

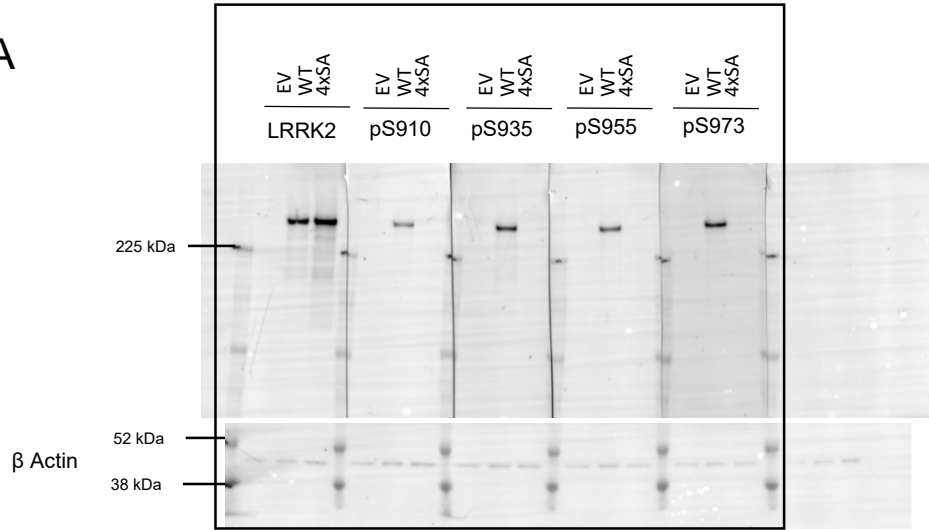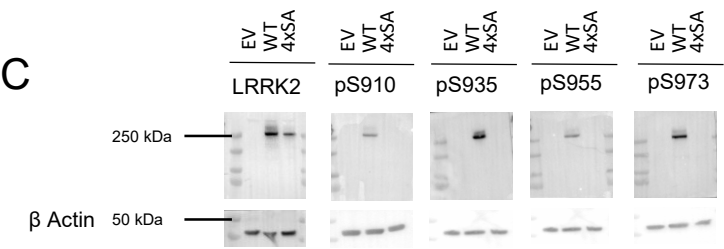

C

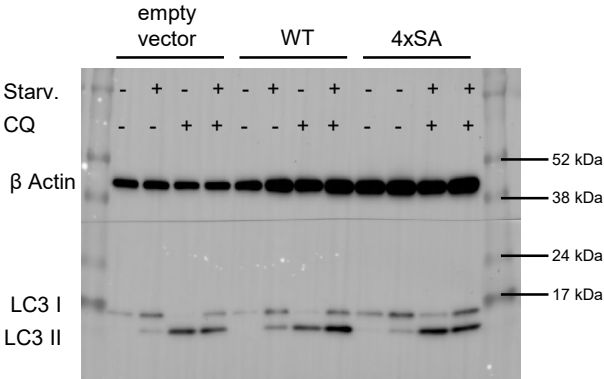

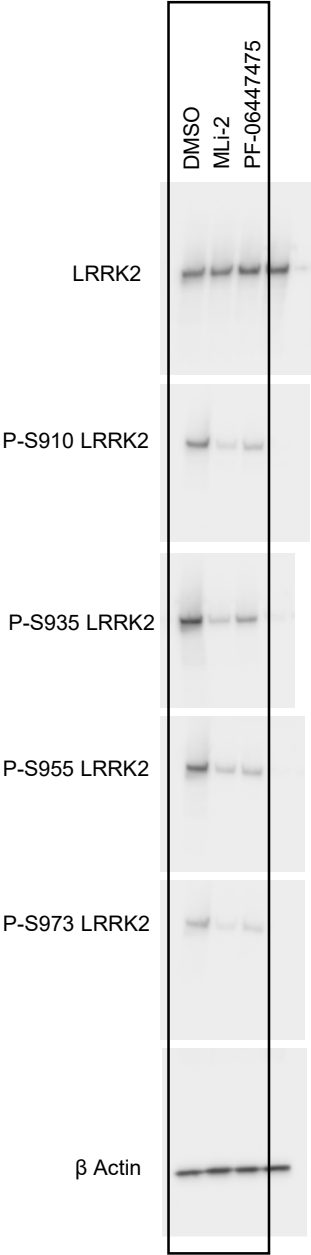

A

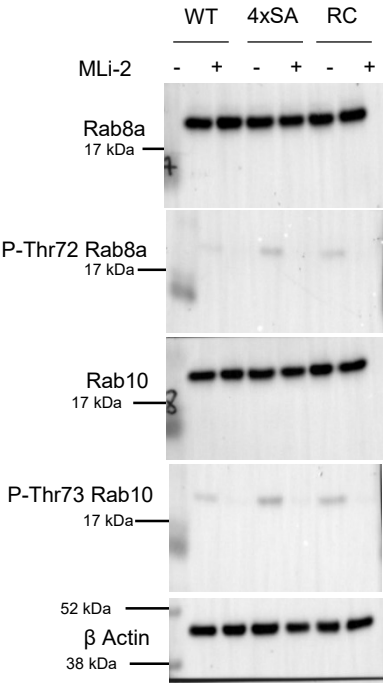

B

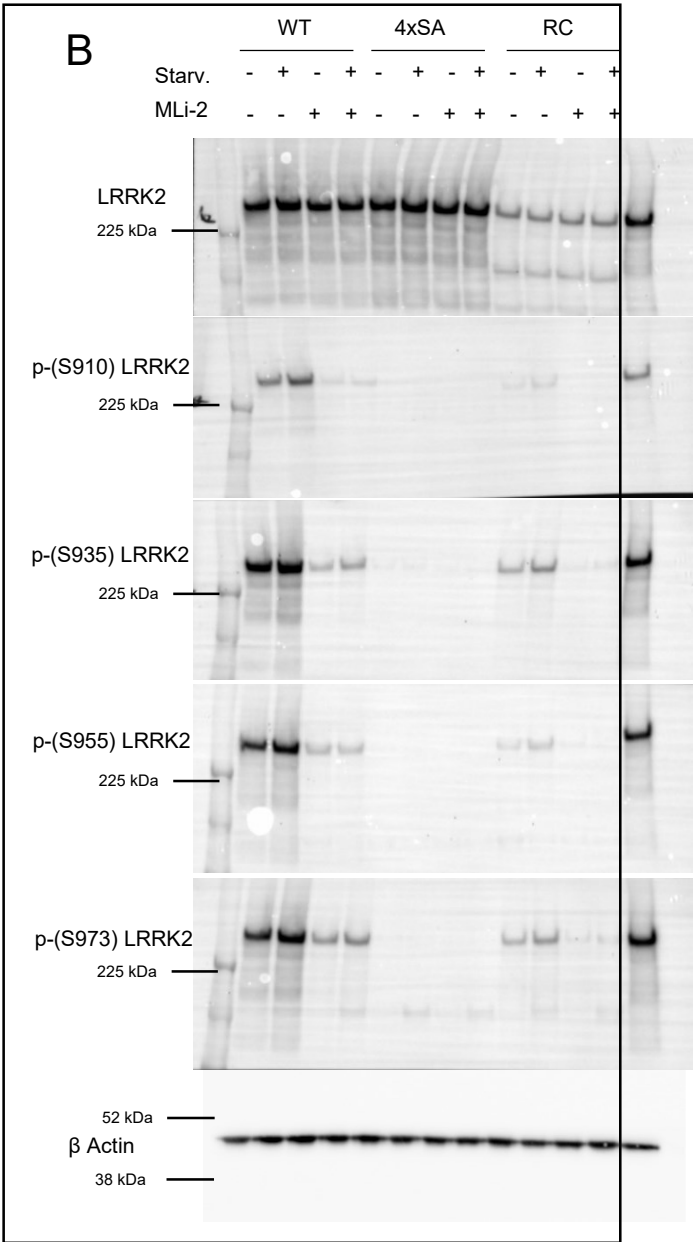

D

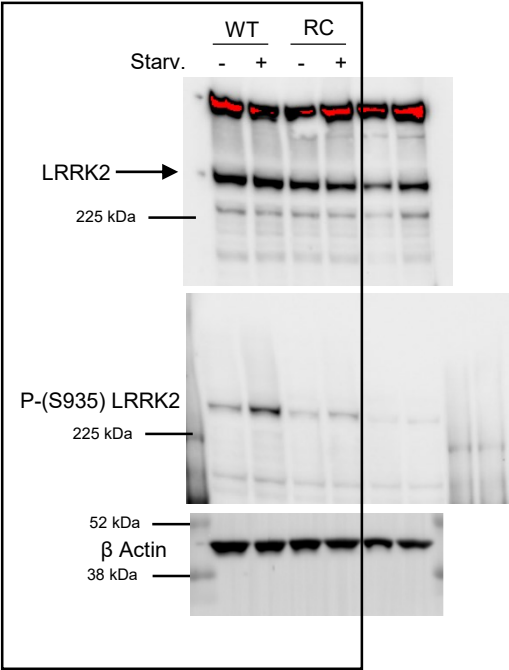

Supplement: Supplementary file 9 — Full-size blots [file 41419_2023_5964_MOESM9_ESM.pdf]
